# A reciprocal regulatory loop between TAZ/YAP and G-protein Gαs regulates Schwann cell proliferation and myelination

**DOI:** 10.1038/ncomms15161

**Published:** 2017-04-26

**Authors:** Yaqi Deng, Lai Man Natalie Wu, Shujun Bai, Chuntao Zhao, Haibo Wang, Jincheng Wang, Lingli Xu, Masahide Sakabe, Wenhao Zhou, Mei Xin, Q. Richard Lu

**Affiliations:** 1 Division of Experimental Hematology and Cancer Biology, Cincinnati Children's Hospital Medical Center, Cincinnati, Ohio 45229, USA; 2 Key Laboratory of Birth Defects and Related Diseases of Women and Children (Ministry of Education), State Key Laboratory of Biotherapy, West China Second Hospital, Sichuan University, Chengdu 610041, China; 3 Institute of Pharmacology and Toxicology, College of Pharmaceutical Sciences, Zhejiang University, Hangzhou 310058, China; 4 Key Laboratory of Birth Defects, Children's Hospital of Fudan University, Shanghai 201102, China

## Abstract

Schwann cell (SC) myelination in the peripheral nervous system is essential for motor function, and uncontrolled SC proliferation occurs in cancer. Here, we show that a dual role for Hippo effectors TAZ and YAP in SC proliferation and myelination through modulating G-protein expression and interacting with SOX10, respectively. Developmentally regulated mutagenesis indicates that TAZ/YAP are critical for SC proliferation and differentiation in a stage-dependent manner. Genome-wide occupancy mapping and transcriptome profiling reveal that nuclear TAZ/YAP promote SC proliferation by activating cell cycle regulators, while targeting critical differentiation regulators in cooperation with SOX10 for myelination. We further identify that TAZ targets and represses *Gnas*, encoding Gαs-protein, which opposes TAZ/YAP activities to decelerate proliferation. *Gnas* deletion expands SC precursor pools and blocks peripheral myelination. Thus, the Hippo/TAZ/YAP and Gαs-protein feedback circuit functions as a fulcrum balancing SC proliferation and differentiation, providing insights into molecular programming of SC lineage progression and homeostasis.

## Abstract (web summary)

The Hippo pathway has recently been implicated in Schwann cell (SC) development and myelination. Here the authors reveal mechanistic insights into how TAZ and YAP regulate and interact with target genes; they further identify a negative feedback loop between TAZ/YAP and G protein Gαs that balances SC proliferation and differentiation.

Schwann cells (SCs) produce multilamellar myelin sheaths that are essential for saltatory conduction of action potentials and axonal integrity in the vertebrate peripheral nervous system (PNS)^1^,^2^,^3^. The mutually exclusive aspects of proliferation and differentiation must be tightly regulated to allow both processes to operate properly to generate sufficient SCs for subsequent differentiation and myelination in developing peripheral nerves. Defects in SC generation and differentiation during development and regeneration may cause a failure in myelinogenesis, contributing to acquired or hereditary peripheral neuropathies associated with motor and sensory disabilities^4^. In contrast, SC over-proliferation, caused by mutations in tumour suppressor genes *NF1* and *NF2*, results in peripheral nerve sheath tumours such as neurofibromas^5^ and Schwannomas^6^, respectively.

SC development is a molecularly and ultrastructurally well-defined, multi-stage process that occurs over a protracted period of time. After specification from neural crest cells and transitioning to a SC precursor state, SC precursors and immature SCs undergo proliferation and expansion to establish a one-to-one relationship with axons during the radial sorting process. Shortly after birth, immature SCs differentiate into pro-myelinating cells and then further differentiate and form myelin sheaths around axons. SC lineage progression requires the coordinated activity of pro-myelinating factors (for example, SOX10, OCT6/POU3F1 and EGR2/KROX20) in promoting cell cycle exit and initiate differentiation, while suppressing negative regulatory factors of differentiation (for example, SOX2, NOTCH/HES and c-JUN)^7^,^8^. At present, the extrinsic and intrinsic signals that modulate and balance positive and negative factors to control SC proliferation and their transition to a differentiating state during peripheral myelination are not fully understood.

Signalling by the tumour suppressor Hippo is an evolutionary conserved pathway that controls organogenesis by regulating cell proliferation, differentiation and, when dysregulated, tumorigenesis^9^,^10^,^11^. Yes-associated-protein (YAP) and transcriptional co-activator with PDZ-binding-motif (TAZ) are the major effectors of Hippo signalling^12^. YAP and TAZ associate with DNA-binding transcription factors, such as TEAD1–4, to regulate downstream gene expression^11^,^13^,^14^. Upstream Hippo kinase cascades phosphorylate and inactivate TAZ/YAP, thereby preventing their nuclear translocation and leading to their ubiquitin-mediated degradation^15^,^16^,^17^,^18^. TAZ/YAP activity can be regulated by multiple signalling pathways, including G-protein coupled receptors (GPCR), Transforming growth factor (TGF-β), wingless/integrated (WNT), and NOTCH, and by mechanical stimuli^19^,^20^,^21^,^22^. Activation of Gαs-protein and cAMP-dependent protein kinase A pathways inhibits YAP activity through preventing its nuclear translocation, whereas elevation of G12/13 or Gαi signalling, which antagonizes the Gαs-mediated cyclic adenosine monophosphate (cAMP) stimulation, enhances YAP expression^19^,^23^. Thus, the activity of TAZ/YAP is tightly regulated in response to context-specific signals^9^,^10^,^24^,^25^. Conversely, regulation of G-protein expression by YAP/TAZ remains elusive.

Recent studies indicate that TAZ/YAP are required for SC radial sorting and peripheral myelination through regulating laminin receptors^22^ or KROX20 (ref. 26), and in control of myelin internodal length^27^. The nuclear localization of YAP/TAZ can be modulated by mechanical signals^22^. YAP/TAZ interact with TEAD1 to regulate expression of myelination-associated genes including *Pmp22* (ref. 28), suggesting that YAP/TAZ may directly regulate the transcriptional programme necessary for SC differentiation. Given that the phenotype of *Taz/Yap* double mutants is much more severe than those observed in mice lacking laminin receptors^29^, it is conceivable that YAP/TAZ regulate additional targets that are responsible for the severe peripheral dysmyelinating phenotype. Currently, the direct targets regulated by YAP/TAZ during SC lineage progression have not been fully defined. Whether YAP/TAZ have a direct role in the transition from SC proliferation-to-differentiation remains unresolved.

Here we show that YAP/TAZ are expressed in SC nuclei in both culture and peripheral nerves through adulthood in mice and demonstrate that YAP/TAZ are crucial for SC proliferation in addition to myelin formation. We further map TAZ genome occupancy in SCs using chromatin immunoprecipitation and sequencing (ChIP-seq) and reveal TAZ direct targets responsible for SC proliferation and differentiation processes. We identify a TAZ target, *Gnas*, encoding Gαs-protein, whereby Gαs functions as a molecular link in a feedback regulation that attenuates TAZ/YAP activity and modulates the transition from SC proliferation to differentiation. These novel mechanistic insights into how TAZ/YAP-mediated Hippo signalling and Gαs-feedback circuits control the opposing aspects of SC proliferation and differentiation provide a rationale for context-specific targeting of TAZ and YAP for treating peripheral neuropathy.

## Results

### Nuclear TAZ and YAP are expressed in Schwann cells

**Figure 1:**
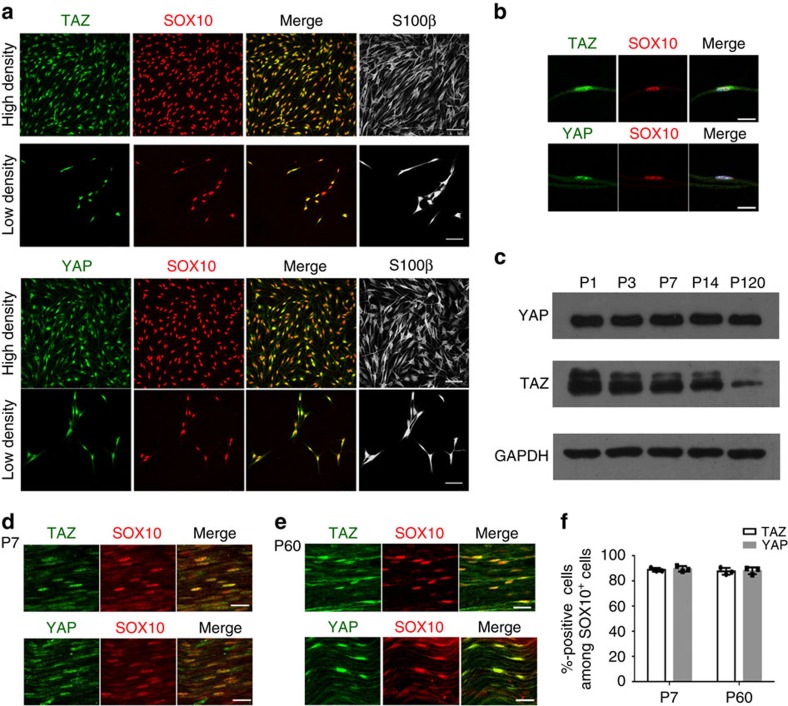
Expression of TAZ and YAP during SC development. (**a**) Immunofluorescence staining of TAZ (upper panels) and YAP (lower panels), SOX10 and S100β (white) in rat SCs at low and high cell densities. Scale bar, 100 μm. (**b**) Immunofluorescence staining of TAZ (green, upper panels) and YAP (green, lower panels) and SOX10 (red) in teased fibres from mouse P8 sciatic nerves. 4,6-Diamidino-2-phenylindole (DAPI) nuclear counterstain is shown in blue. Scale bar, 10 μm. (**c**) Western blot of TAZ and YAP of sciatic nerves at developmental time points as indicated (The experiment was repeated twice on 2 animals). (**d**) Immunofluorescence staining of TAZ (green, upper panels) and YAP (green, lower panels) and SOX10 (red) in a sciatic nerve section at P7. Scale bar, 20 μm. (**e**) Immunofluorescence staining of TAZ (green, upper panels) and YAP (green, lower panels) and SOX10 (red) in a sciatic nerve section at P60. Scale bar, 20 μm. (**f**) Percentages of cells expressing TAZ or YAP among SOX10^+^ SCs in wild-type mice at P7 and P60. (*n*=3 animals/genotype; the experiment was repeated on 3 different sections/animal).

The subcellular distribution of TAZ/YAP can be modulated by various extrinsic signals such as cell density^14^,^16^. To examine expression and subcellular localization of TAZ and YAP in SCs, we performed co-immunolabelling with TAZ or YAP and SC markers, nuclear SOX10 and cytoplasmic S100β, in rat SCs. Both TAZ and YAP were predominantly detected in the nuclei of SCs at both low- and high-cell density (Fig. 1a). Similar nuclear localization of TAZ or YAP was also observed in SCs in the teased fibres from mouse sciatic nerves at P8 (Fig. 1b). YAP expression was abundant throughout development (Fig. 1c), and the level of the phosphorylated YAP did not exhibit substantial change at different postnatal stages (Supplementary Fig. 1). In contrast, TAZ expression was dynamically regulated and was gradually downregulated in adulthood (Fig. 1c). In sciatic nerves, persistent nuclear TAZ/YAP localization was observed in SCs at the neonatal stages, for example, P7 (Fig. 1d) and even in fully myelinated adult stages for example, at P60 (Fig. 1e). The majority (∼85%) of cells in the sciatic nerves that expressed YAP or TAZ were also SOX10^+^ (Fig. 1f), suggesting an active transcriptional regulatory role of TAZ/YAP in SC development and myelination.

### *Taz* and *Yap* inactivation disrupts SC development

**Figure 2:**
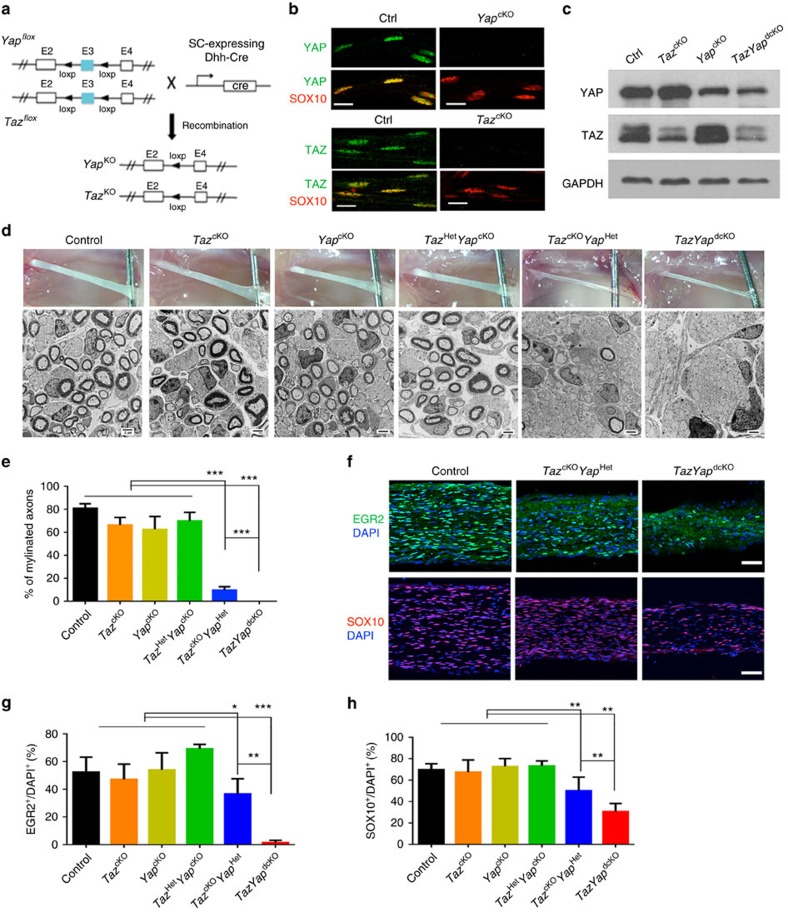
Mice with *Taz*and*Yap* deletion exhibit myelination defects in sciatic nerves. (**a**) Schematic diagram shows that exon 3, flanked by *loxP* sites, of the floxed *Taz* or *Yap* alleles is excised upon recombination mediated by SC-expressing *Dhh-Cre*. (**b**) Immunofluorescence staining of YAP (green, upper panels), TAZ (green, lower panels) and SOX10 (red) in teased fibres of wild-type control and *Taz*^cKO^ or *Yap*^cKO^ sciatic nerves at P7. Scale bar, 10 μm. (**c**) Western blot for TAZ and YAP in sciatic nerves from control and *Taz*^cKO^, *Yap*^cKO^ and *TazYap*^dcKO^ mice at P7 (The experiment was repeated twice on 2 animals). (**d**) Appearance of sciatic nerves (upper panels) and electron microscopy of sciatic nerve ultrastructure (lower panels) in age-matching wild-type control and indicated *Taz* and *Yap* mutants at P7. Scale bar, 2 μm. (**e**) Quantification of percentage of myelinated axons in control and *Taz* and *Yap* mutant sciatic nerves at P7 (*n*=5 animals/genotype). Plotted are means±s.e.m. ***P<0.001; one-way ANOVA with Tukey's multiple-comparison test. (**f**) Immunofluorescence labelling for EGR2 (green), SOX10 (red) and DAPI (blue) in P7 sciatic nerves of control and *Taz* and *Yap* mutants at P7. Scale bar, 50 μm. (**g**,**h**) Percentage of (**g**) EGR2-expressing cells and (**h**) SOX10-expressing cells among DAPI^+^ SCs in control and *Taz* and *Yap* mutants at P7 (*n*=5 animals/genotype; the experiment was repeated on 3 different sections/animal). Plotted are means±s.e.m. *P<0.05; **P<0.01; ***P<0.001; one-way ANOVA with Tukey's multiple-comparison test.

To define the cell-autonomous role of TAZ/YAP in SC development, we bred mice carrying a *Yap*-floxed allele (*Yap*^fl/fl^), a *Taz*-floxed allele (*Taz*^fl/fl^) or both^30^ with desert-hedgehog (Dhh)-Cre mice^31^ (Fig. 2a), where Cre-mediated recombination is active in the SC lineage from embryonic day 12.5 (E12.5). These crosses generated *Taz*^fl/fl^; *Dhh-Cre* mice (referred to here as *Taz*^cKO^), *Yap*^fl/fl^;*Dhh-Cre* mice (*Yap*^cKO^) and the double mutants *Yap*^fl/fl^;*Taz*^fl/fl^;*Dhh-Cre* (*TazYap*^dcKO^). That TAZ or YAP protein expression was efficiently abolished in SOX10^+^ SCs of developing sciatic nerves was verified by immunostaining of teased nerve fibres (Fig. 2b) and western blot analysis of sciatic nerve tissues (Fig. 2c). We detected upregulation of YAP or TAZ protein expression in *Taz* or *Yap* single-mutant sciatic nerves, respectively (Fig. 2c), suggesting a compensatory effect in each other's absence. Both *Taz*^cKO^ and *Yap*^cKO^ mice appeared phenotypically normal as compared to heterozygous control littermates at P60. At P7, sciatic nerves in these mutants were thick and opaque, and comparable to wild-type controls (Fig. 2d). Ultrastructural analysis of sciatic nerves from *Taz*^cKO^ and *Yap*^cKO^ mice had the expected 1:1 ratio of SC to axons and showed no significant impairments in myelination (Fig. 2d,e). Total SOX10^+^ SCs and EGR2^+^ myelinating SCs in *Taz*^cKO^ and *Yap*^cKO^ mice were unaltered relative to wild-type controls (Fig. 2f–h). The absence of apparent myelination defects in *Taz* or *Yap* single-mutants suggests that TAZ and YAP function redundantly during SC development.

We then analysed the double mutant *TazYap*^dcKO^ and *Taz*^cKO^ heterozygous for *Yap* (*Taz*^fl/fl^; *Yap*^fl/+^; *Dhh-Cre*, referred to as *Yap*^cKO^*Taz*^Het^) and *Yap*^cKO^ heterozygous for *Taz* (*Yap*^fl/fl^; *Taz*^fl/+^; *Dhh-Cre*, referred to as *Taz*^cKO^*Yap*^Het^) to investigate dosage-dependent effects of TAZ/YAP on SC development. The *Yap*^cKO^*Taz*^Het^ mice were phenotypically indistinguishable from control littermates with properly sorted and myelinated large calibre axons at P7 (Fig. 2d,e). In contrast, *Taz*^cKO^*Yap*^Het^ sciatic nerves appeared thin and translucent and had radial sorting and myelination deficiencies (Fig. 2d,e and Supplementary Fig. 2). In addition, SOX10^+^ SCs and EGR2^+^ SCs were reduced in number in *Taz*^cKO^*Yap*^Het^ mice (Fig. 2f–h). Consistent with P0-Cre-mediated *Taz/Yap* mutations^22^, one allele of *Yap* is not sufficient to compensate for *Taz* deficiency, suggesting that TAZ plays a more dominant role than YAP in SC development.

Strikingly, *TazYap*^dcKO^ double mutant mice developed severe tremors and hindlimb paralysis and died around postnatal week 3. Concurrent *Taz*/*Yap* loss in SCs further exacerbated the radial sorting defects, as demonstrated by the presence of large bundles of unsorted axons and a remarkable reduction in the number of SOX10^+^ SC lineage cells and EGR2^+^ differentiating SCs relative to controls, *Taz* or *Yap* single mutants as well as *Taz*^cKO^*Yap*^Het^ mice (Fig. 2f–h; and Supplementary Fig. 2). The majority of *TazYap*^dcKO^ SCs failed to develop beyond the immature stage, as revealed by the absence of myelinating profiles (Fig. 2d,e), indicating that *Taz*/*Yap* are required for SC lineage progression and subsequent myelination.

### TAZ and YAP are critical for SC proliferation

**Figure 3:**
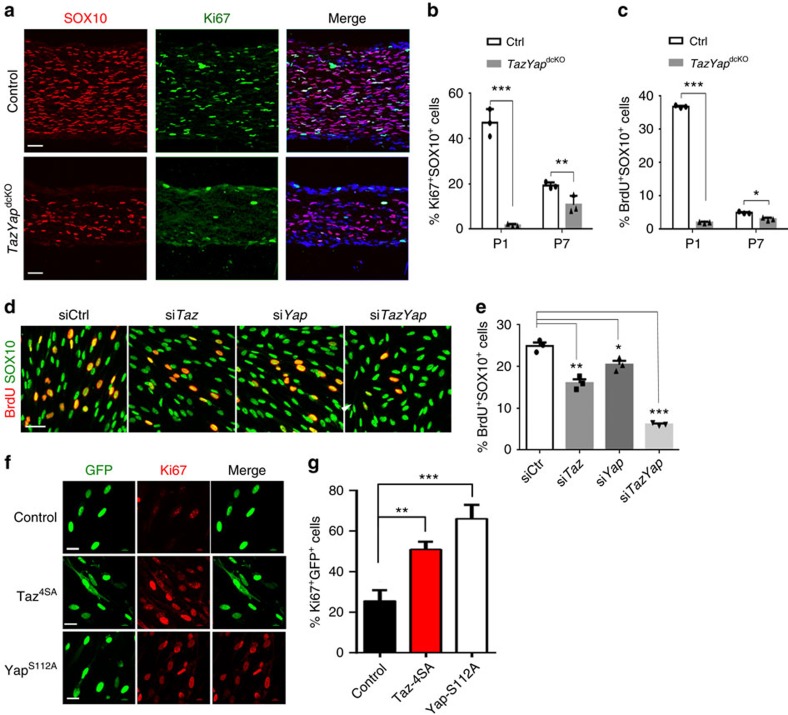
TAZ and YAP regulate SC proliferation *in vivo* and *in vitro*. (**a**) Immunofluorescence labelling for SOX10 (red) and Ki67 (green) in sciatic nerves of wild-type control and *TazYap*^dcKO^ mice at P7. Nuclei were counterstained with DAPI. Scale bar, 50 μm. (**b**,**c**) Quantification of (**b**) Ki67^+^ and (**c**) BrdU^+^ cells among SOX10^+^ SCs in sciatic nerves of control and *TazYap*^dcKO^ mice at P1 and P7 (*n*=3 animals/genotype; the experiment was repeated on 3 different sections/animal). Data are means±s.e.m. ***P<0.001; Two-tailed unpaired Student's *t-*test. (**d**) Immunofluorescence labelling for BrdU (red) and SOX10 (green) in SCs transfected with control siRNA or siRNAs targeting *Taz, Yap*, or both. Scale bar, 50 μm. (**e**) Quantification of proliferation of SCs transfected with control siRNA or siRNA targeting *Taz, Yap*, or both (*n*=5 independent experiments). Data plotted are means±s.e.m. *P<0.05, **P<0.01, ***P<0.001; one-way ANOVA with Tukey's multiple-comparison test. (**f**) Immunofluorescence labelling for Ki67 (red) in SCs transfected with control vector (GFP only) or Taz-4SA or Yap-S112A expressing vectors. Scale bar, 20 μm. (**g**) Quantification of Ki67^+^ expression in SCs transfected with control vector (GFP only) or Taz-4SA or Yap-S112A expressing vectors. The experiment was repeated 5 times. Data are means±s.e.m. **P<0.01, ***P<0.001; one-way ANOVA with Tukey's multiple-comparison test.

SCs must proliferate to match axon number for radial sorting. The severe reduction in SC number in *TazYap*^dcKO^ sciatic nerves prompted us to hypothesize that the arrest of SC lineage progression when TAZ/YAP are lost is a consequence of defects in SC proliferation and expansion. We quantified SC proliferation in sciatic nerves in SOX10^+^ SCs by Ki67 immunolabelling and bromodeoxyuridine (BrdU) incorporation at P1 and P7, and observed a stark reduction in Ki67-expressing and BrdU-labelling SCs in *TazYap*^dcKO^ mice (Fig. 3a–c). We did not detect significant differences in cell death in the *TazYap*^dcKO^ mice (Supplementary Fig. 3), suggesting that both *Taz/Yap* are required for SC proliferation.

To further investigate the function of *Taz/Yap* in SC proliferation, we inhibited expression of *Taz* or *Yap*, or both using siRNA in purified rat SCs under growth conditions. Reduced expression of *Taz*- or *Yap*-decreased SC proliferation as assayed by BrdU incorporation (Fig. 3d,e). Inhibition of both *Taz* and *Yap* resulted in more severely compromised SC proliferation than when expression of a single factor was inhibited (Fig. 3d,e). Conversely, expression of constitutively active form of TAZ (Taz^4SA^ with serine-to-alanine mutations of four phosphorylation sites)^32^, or YAP (Yap^S112A^ with mutation of the phosphorylation site serine 112 to alanine)^33^, robustly enhanced SC proliferation (Fig. 3f,g). Together, our data indicate that activation of TAZ/YAP promotes SC proliferation, consistent with their pro-growth and oncogenic function in other contexts^34^.

### TAZ/YAP control the initiation of SC differentiation

**Figure 4:**
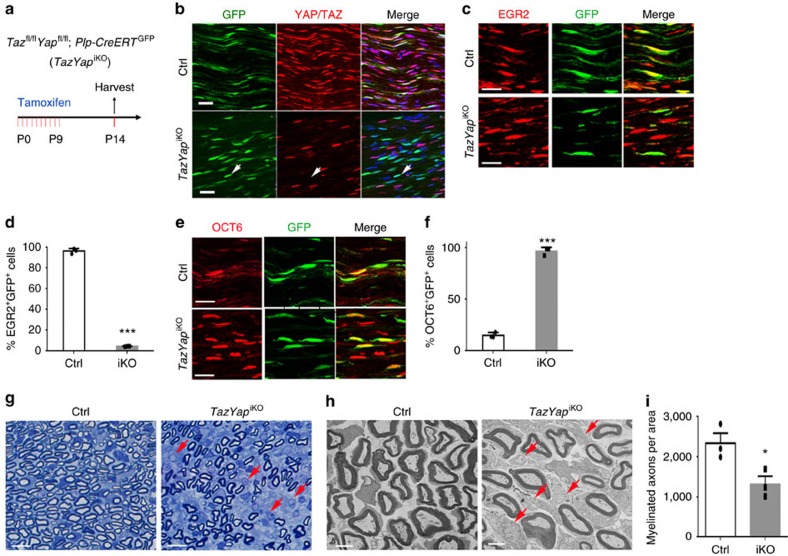
YAP and TAZ are required for the initiation of SC differentiation. (**a**) Schematic representation of tamoxifen administration to *TazYap*^iKO^ mice. Mice were treated with 1 mg per 10 g body weight tamoxifen daily from P0 to P9. Sciatic nerves were harvested at P14. (**b**) Immunofluorescence labelling of GFP and anti-YAP/TAZ antibodies in nerves from control (*Plp*-*CreERT;*^*cGFP*^) and *TazYap*^iKO^ mice at P14. Arrows indicate a GFP^+^ and YAP/TAZ^−^ cell. Scale bars, 20 μm. (**c**) Immunofluorescence labelling of EGR2^+^ (red) and GFP^+^ (green) cells in nerves from control (*Plp*-*CreERT*;^cGFP^) and *TazYap*^iKO^ mice at P14. Scale bars, 20 μm. (**d**) Quantification of EGR2^+^ among GFP^+^ cells in control and *TazYap*^iKO^ nerves at P14 (*n*=3 animals/genotype; the experiment was repeated 3 times). Data are means±s.e.m. ***P<0.001; Two-tailed unpaired Student's *t-*test. (**e**) Immunofluorescence labelling of OCT6^+^ (red) and GFP^+^ (green) cells in control and *TazYap*^iKO^ nerves at P14. Scale bars, 20 μm. (**f**) Quantification of OCT6^+^ among GFP^+^ cells in control and *TazYap*^iKO^ nerves at P14 (*n*=3 animals/genotype; the experiment was repeated 3 times). Data plotted are means±s.e.m. ***P<0.001; Two-tailed unpaired Student's *t-*test. (**g**) Thick section of cross-sections of sciatic nerves from control and *TazYap*^iKO^ nerves at P14. Arrows denote unmyelinated axons. Scale bar, 4 μm. (**h**) Electron micrographs of cross-sections of sciatic nerves from control and *TazYap*^iKO^ nerves at P14. Arrows denote unmyelinated axons. Scale bars, 2 μm. (**i**) Quantification of myelinated axon number per cross-section of sciatic nerves from control and *TazYap*^iKO^ nerves at P14 (*n*=3 animals/genotype; the experiment was repeated on at least five different sections/animal). Data are presented as means±s.e.m. *P<0.05; Two-tailed unpaired Student's *t-*test.

To determine the role of TAZ/YAP in the differentiation process, we inactivated *Taz/Yap* in immature SCs in neonates by using a tamoxifen-inducible SC-expressing *Plp*-*CreERT* driver^35^ carrying a floxed CAG-GFP (green fluorescence protein) reporter (ccGFP)^36^ to bypass the possible impact of the *Taz/Yap* loss on early SC development. Mice were treated with tamoxifen from P0 to P9 to induce deletion of *Taz* and *Yap*; we refer to these mice as *TazYap*^iKO^ mice (Fig. 4a). TAZ/YAP were efficiently depleted in GFP reporter-expressing cells after tamoxifen treatment (Fig. 4b). We detected a severe reduction in the percentage of EGR2^+^ mature SCs while an increase in the percentage of the immature OCT6^+^ SCs with *Taz/Yap* deletion (Fig. 4c–f). Compared to control nerves at P14, about 42% of the large axons were not properly sorted and remained unmyelinated in *TazYap*^iKO^ nerves (Fig. 4g–i), suggesting that YAP/TAZ are required for proper initiation of SC differentiation from immature SCs and their subsequent myelination. Intriguingly, tamoxifen-induced deletion of *Taz/Yap* in adult mice did not substantially alter myelin sheath thickness nor the integrity in peripheral nerves (Supplementary Fig. 4), despite the fact that these mice exhibited severe tremors, ataxia and finally mortality within one month after tamoxifen-induced *Taz* and *Yap* deletion. These observations suggest a crucial role for TAZ/YAP in initiating SC differentiation. There appears to be no immediate requirement for YAP/TAZ for peripheral myelination maintenance, however, due to lethality we were unable to examine mice more than a month post-ablation.

### TAZ/YAP regulate SC proliferation and myelination programs

**Figure 5:**
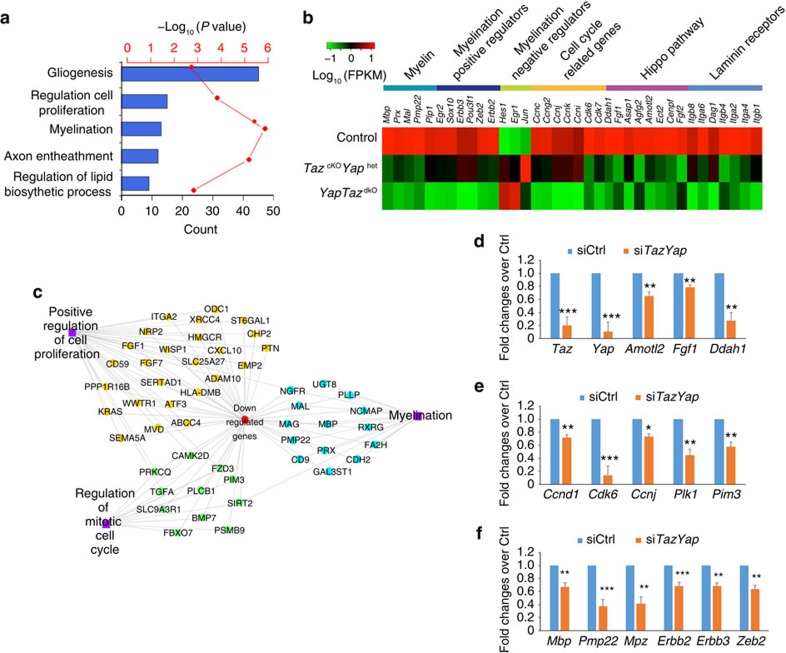
YAP and TAZ regulate expression of the network of myelination and proliferation-related genes. (**a**) Gene ontology (GO) analysis of genes downregulated in *TazYap*^dcKO^ sciatic nerves compared to control nerves. (**b**) Heatmap of the average expression levels of myelination and proliferation-associated genes, Hippo-pathway downstream genes, and laminin receptor genes in sciatic nerves of wild-type control, *Taz*^cKO^*Yap*^Het^ and *TazYap*^dcKO^ mice at P5. (**c**) ToppCluster plot showing the functional networks among the genes downregulated in *TazYap*^dcKO^ sciatic nerves. (**d**–**f**) qRT-PCR analyses of Hippo-downstream genes (**d**), cell proliferation-related genes (**e**) and myelination-associated genes (**f**) following treatments with scrambled siRNA control or siRNAs targeted *Taz* and *Yap*. Data are presented as means±s.e.m.; *n*=5 independent experiments; the experiment was repeated 5 times. *P<0.05, **P<0.01, ***P<0.001; one-way ANOVA with *post hoc* Tukey's test.

Next, we sought to define the underlying mechanisms whereby TAZ/YAP regulate SC growth and differentiation by comparing the transcriptome profiles of P5 sciatic nerves consisting of proliferating and differentiating SCs from control, *Taz*^cKO^*Yap*^Het^, and *TazYap*^dcKO^ mice. Consistent with the intermediate dysmyelinating phenotype of *Taz*^cKO^*Yap*^Het^ mice, the changes in the expression pattern were intermediate between control and *TazYap*^dcKO^ mice. We therefore focused our comparison on the transcriptomes of control and *TazYap*^dcKO^ mice. Gene ontology analysis revealed that the downregulated genes in *TazYap*^dcKO^ mice were enriched for cell proliferation regulation, lipid biosynthesis and myelination (Fig. 5a,b), consistent with deficient SC proliferation and the dysmyelination phenotype in these mutants.

Among the differentially expressed genes, we identified a set of Hippo pathway signatures related to cell growth, including *Amotl2, Fgf1, Ect2* and *Ddah1* (refs 37, 38; Fig. 5b), that were downregulated in *TazYap*^dcKO^, consonant with TAZ/YAP as the key effectors in Hippo signalling. In addition, expression of critical myelination regulators such as *Egr2* and *Zeb2/Sip1*, as well as myelin genes, *Mbp, Pmp22* and *Erbb2/3*, were dramatically downregulated in *TazYap*^dcKO^ nerves, with a concomitant elevation of myelination inhibitory genes such as *Hes1* and *Egr1* (Fig. 5b; Supplementary Table 1). In accordance with the reduction of SC number, we detected a decrease in a set of cell cycle-related genes *Ccnj, Cdk6, Pim3* and *Mycn* (Fig. 5b). Consistent with severe radial sorting defect in the *TazYap*^dcKO^ nerves, expression of genes encoding critical regulators of radial sorting including *Dag1, Itgb1, Itga6* and *Itga8* (ref. 22) was reduced (Fig. 5b). In agreement, analysis of the global gene regulatory network also showed dysregulation of genes involved in cell proliferation, mitotic cell cycle and in myelination in *TazYap*^dcKO^ nerves (Fig. 5c). Since the apparent gene expression changes might reflect a lower proportion of SCs in *TazYap*^dcKO^ nerves, to investigate whether TAZ/YAP regulate gene expression programs of SC proliferation and differentiation in a cell-autonomous manner, we carried out siRNA-mediated knockdown of *Taz* and *Yap* in rat SCs. Expression of Hippo-downstream targets (Fig. 5d) and cell cycle/proliferation-related genes (Fig. 5e), as well as SC differentiation-associated genes (Fig. 5f) was significantly reduced in SCs that underwent *Taz* and *Yap* knockdown. Collectively, these data suggest that TAZ/YAP control regulatory networks for SC proliferation and differentiation.

### TAZ cooperates with SOX10 to regulate SC differentiation

**Figure 6:**
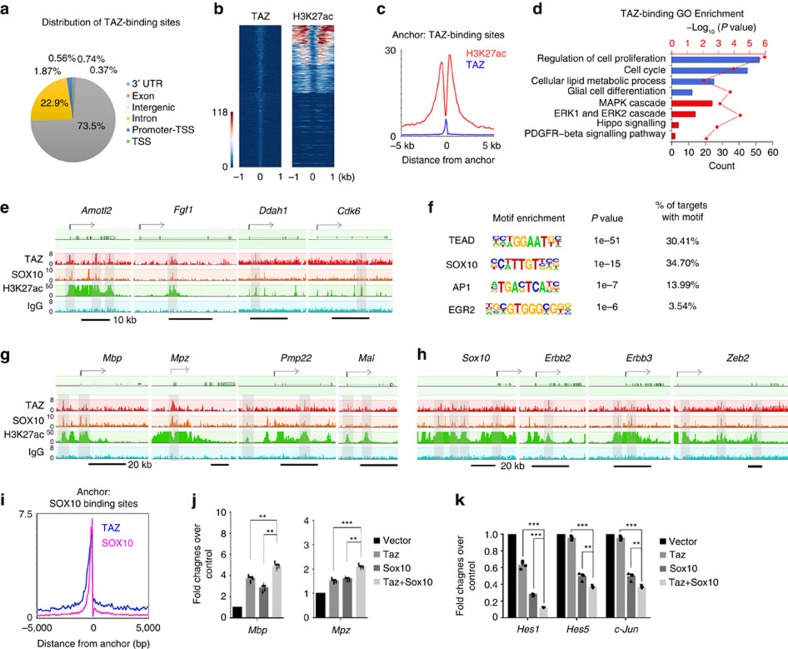
TAZ targets the enhancers of SC proliferation and differentiation genes and interacts with SOX10. (**a**) Pie chart showing genomic global distribution of TAZ-binding sites. (**b**) ChIP-seq density heatmaps for TAZ and H3K27Ac within ±1 kb of the TAZ peak center. (**c**) Average H3K27Ac enrichment profiles around the central position of TAZ-binding regions. (**d**) GO analysis of TAZ-binding genes. (**e**) ChIP-seq binding peaks of TAZ and H3K27Ac from rat SCs and SOX10 from sciatic nerves on the gene loci associated with the Hippo pathway and cell proliferation. Grey-shaded regions highlighted binding peaks. (**f**) Homer motif enrichment analysis of the TAZ-binding cistrome in rat SCs. (**g**,**h**) ChIP-seq binding peaks of TAZ and H3K27Ac from rat SCs and SOX10 from sciatic nerves on the gene loci associated SC myelin genes (**g**), and myelination regulators (**h**). Grey shaded regions highlighted binding peaks. (**i**) Average SOX10 ChIP-seq enrichment profiles around the central position of TAZ-binding regions. (**j**,**k**) Luciferase activity of reporters driven by promoters of *Mbp*, *Mpz* (**j**) and *Hes1, Hes5* and *c-Jun* (**k**) in 293T cells co-transfected with control vector or vectors for expression of TAZ or both TAZ and SOX10. Values presented are the average of three independent experiments (*P<0.05; **P<0.01, ***P<0.001; one-way ANOVA with *post hoc* Tukey's test).

Given that TAZ/YAP have similar genomic binding profiles^39^, to gain insights into TAZ/YAP target genes, we performed ChIP-seq analysis in primary rat SCs to assess the chromatin occupancy of TAZ, which has a more prominent role than YAP in SC development. We analysed the distribution of TAZ-binding regions and found that a large proportion of TAZ-binding peaks (∼73%) mapped to the evolutionarily conserved enhancer elements in promoter and intergenic regions (Fig. 6a), consistent with previous reports in other contexts^39^. These peaks were associated with an activating histone mark H3K27Ac (Fig. 6b), and a bimodal distribution of H3K27Ac around TAZ peaks was observed (Fig. 6c). Gene ontology revealed that these TAZ direct targets are associated with biological processes related to cell proliferation, as well as glial/SC differentiation (Fig. 6d). TAZ occupancy was detected around the promoter and enhancer regions of cell growth-related canonical Hippo pathway genes including *Amotl2, Fgf1* and *Ddah1* as well as cell cycle regulators including *Cdk6* (Fig. 6e). Accompanying the activating H3K27ac mark, TAZ occupancy likely activates expression of these cell proliferation-related genes, consistent with downregulation of their expression in *TazYap* knockout nerves or knockdown SCs (Fig. 5).

To further explore the potential co-regulators of TAZ, we analysed the potential transcriptional co-regulator binding motifs enriched in TAZ-targeted sites using HOMER motif-discovery algorithm^40^. Motif analysis revealed a strong enrichment of the known TEAD-binding consensus motif GGAATG^41^ within TAZ-binding sites (Fig. 6f), suggesting that TAZ exerts its transcriptional control via TEAD, consistent with a previous study of YAP genomic occupancy^41^.

In addition to the TEAD-consensus motif, we also found that ∼34% of TAZ-binding sites were enriched with the Sox-consensus binding motif C(T/A)TTG(T/A)(T/A) and matched most closely to that of SOX10 (ref. 42), a critical factor for SC lineage progression^3^. Further, there was enrichment for the AP-1 (c-Jun/c-Fos complex) motif TGACTCA, consistent with a previous report in other cellular contexts^39^ (Fig. 6f). This suggests that TAZ/YAP/TEAD cooperate with distinct co-regulators to modulate the gene programs necessary for SC lineage progression.

Analysis of TAZ-binding elements together with SOX10 binding sites in rat sciatic nerves^42^ revealed that TAZ co-occupation with SOX10 at the H3K27ac-marked promoters/enhancers of a set of myelination-associated genes including *Mbp, Mpz, Pmp22* and *Mal,* as well as SC differentiation regulators *Sox10*, *Erbb2/3* and *Zeb2* (Fig. 6g–i). Consistently, TAZ and SOX10 overexpression in combination enhanced the activity of reporter genes driven by the promoters of myelin genes *Mbp* and *Mpz,* which are targeted by SOX10 and TAZ (Fig. 6j), while suppressing those of SC differentiation inhibitors *Hes1, Hes5* and *c-Jun* (Fig. 6k), suggesting that TAZ cooperates with SOX10 to promote the transcriptional programme required for SC differentiation.

### TAZ regulates G-protein signalling

**Figure 7:**
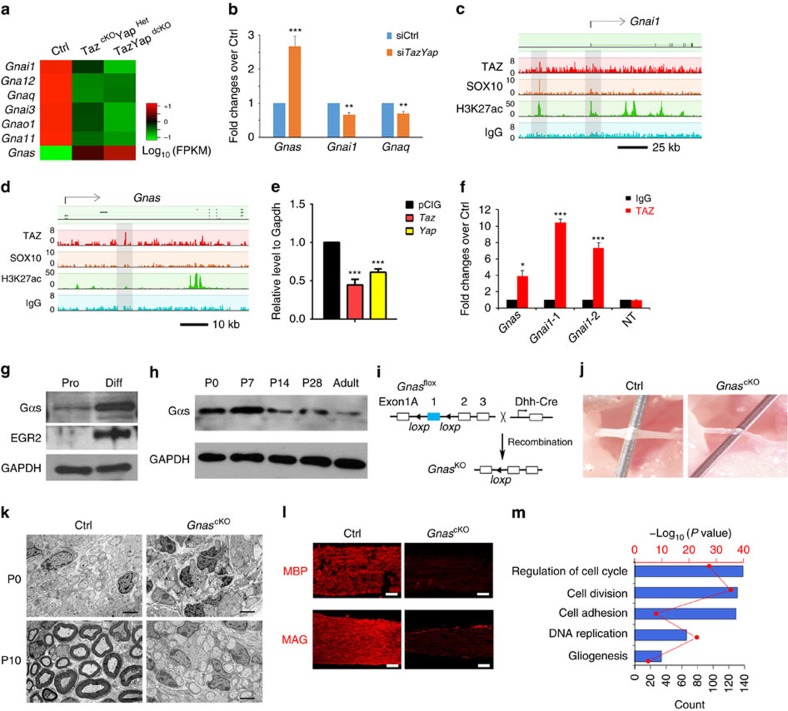
TAZ controls the proliferation of SCs by regulating G-protein signalling. (**a**) Heatmap of the relative levels of G protein-encoding genes by RNA-seq in wild-type control, *Taz*^cKO^*Yap*^Het^ and *TazYap*^dcKO^ sciatic nerves at P5. (**b**) qRT-PCR analyses of G-protein genes following treatments with siRNA control or si*Taz* and si*Yap*. Data are presented as means±s.e.m.; *n*=5 independent experiments, **P<0.01, ***P<0.001; Two-tailed unpaired Student's *t-*test. (**c**,**d**) ChIP-seq binding peaks of TAZ, SOX10 and H3K27Ac on the *Gnai1* (**c**) and *Gnas* (**d**) loci in the genome of rat SCs. Grey-shaded regions highlighted TAZ-binding peaks. (**e**) qRT-PCR quantification of *Gnas* in SCs after transfection with control (pCIG) and TAZ or YAP-expressing vectors. Data are presented as means±s.e.m.; *n*=5 independent experiments, ***P<0.001; one-way ANOVA with *post hoc* Tukey's test. (**f**) TAZ occupancy on TAZ-binding elements on *Gnas* and *Gnai1* loci by ChIP-qPCR using anti-TAZ or IgG control. NT: the non-target control segment lacking TAZ-binding. Data are presented as means±s.e.m.; *n*=5 independent experiments, *P<0.05; ***P<0.001; Two-tailed unpaired Student's *t-*test. (**g**) Western blot of Gαs and EGR2 in SCs under proliferation (Pro) and differentiation (Diff) conditions. GAPDH as a loading control (*n*=3 independent experiments). (**h**) Western blot of Gαs in the sciatic nerves at the indicated stages. GAPDH as a loading control (the experiment was repeated twice on 2 animals). (**i**) Schematic diagram shows that the floxed *Gnas* exon 1 allele is excised upon recombination mediated by *Dhh-Cre*. (**j**) Appearance of sciatic nerves from control (*Gnas*^*fl/+:*^*Dhh-Cre*) and *Gnas*^cKO^ mutants at P10. (**k**) Electron microscopy of sciatic nerve cross-sections from control and *Gnas*^cKO^ mutants at P0 and P10. Scale bars: 1 μm. (**l**) Immunofluorescence of MBP and MAG in control and *Gnas*^cKO^ sciatic nerves at P7. Scale bars: 100 μm. (**m**) Gene ontology analysis of the significantly upregulated genes in *Gnas*^cKO^ compared to control.

Combinatorial analysis of transcriptome profiles and TAZ genome-wide occupancy revealed a set of G-protein signalling genes that were TAZ-occupied and dysregulated in *Taz*/*Yap*-deficient SCs (Fig. 7a). Consistent with their role as agonists of TAZ/YAP activation^43^, the genes encoding Gα proteins including *Gnai1, Gnaq, Gnao1* and *Gna11* were downregulated in *TazYap*^dcKO^ and *Taz*^cKO^*Yap*^Het^ nerves (Fig. 7a). In contrast, *Gnas*, which encodes Gαs, was upregulated in *Taz*/*Yap* mutant nerves (Fig. 7a). Consistently, siRNA knockdown of *Taz*/*Yap* in SCs resulted in an upregulation of *Gnas*, while inhibiting expression of *Gnai1* and *Gnaq* (Fig. 7b).

TAZ and SOX10 co-occupancy was detected in the enhancer region of *Gnai1* in association with H3K27Ac, suggesting that TAZ and SOX10 co-targeting enhances expression of *Gnai1*, which encodes an inhibitory Gαi-protein^19^ (Fig. 7c). In contrast, TAZ targeted to the *Gnas* gene locus was not associated with enrichment of H3K27Ac or SOX10 (Fig. 7d). This prompted us to hypothesize that TAZ represses *Gnas* transcription. Indeed, constitutive expression of TAZ or YAP in rat SCs suppressed *Gnas* expression (Fig. 7e). The enrichment of TAZ occupancy on the targeted elements of *Gnas* and *Gnai1* loci was further confirmed ChIP-qPCR in rat SCs (Fig. 7f). These observations suggest that TAZ directly targets and surprisingly inhibits *Gnas* expression, while activating *Gnai1* expression, indicating a context-specific TAZ activity.

We detected an increase in *Gnas*-encoded Gαs protein expression in SCs cultured under differentiation compared to proliferation conditions (Fig. 7g). Similarly, expression of Gαs protein was detected at the neonatal stage P0, when immature SCs undergo proliferation, and peaked at the perinatal stage P7, the onset of SC myelination, but then subsided in late postnatal stages and adulthood (Fig. 7h). To examine the function of *Gnas* in SC development in vivo, we specifically ablated *Gnas* in SCs using *Dhh-Cre* (Fig. 7i). *Gnas*^cKO^ mice were born at a Mendelian frequency but died around postnatal week 2. *Gnas*^cKO^ mice exhibited severely impaired movement characterized by paralyzed hindlimbs and tremors, similar to the phenotype of *TazYap*^dcKO^ mice. In contrast to controls, *Gnas*^cKO^ sciatic nerves appeared thin and translucent (Fig. 7j), indicative of a myelination deficiency. At P0, when SCs had started to establish a 1:1 relationship with axons in control nerves, a majority of SCs in *Gnas*^cKO^ mice remained associated with multiple large calibre axons (Fig. 7k), suggesting incomplete radial sorting. At P10, control SCs had advanced to myelinate segregated large diameter axons; however, in *Gnas*^cKO^ mice, SCs were essentially arrested at the pro-myelinating stage and failed to initiate myelin sheaths (Fig. 7k). Large unsorted axon bundles were also frequently detected in *Gnas*^cKO^ sciatic nerves (Fig. 7k; Supplementary Fig. 5). The severe myelinogenesis defect in *Gnas*^cKO^ mice was corroborated by the absence of myelin proteins such as MBP and MAG in *Gnas-*mutant nerves (Fig. 7l).

As TAZ directly targets the *Gnas* locus and inhibits its expression, the observation that *Gnas*^cKO^ nerves exhibited a similar morphological phenotype to *TazYap*^dcKO^ nerves appeared to be counterintuitive since both *Gnas* and *Taz/Yap* are needed for SC differentiation. We therefore analysed gene expression profiles of *Gnas*^cKO^ sciatic nerves. Strikingly, gene ontology analysis reveals that the genes upregulated *Gnas*^cKO^ nerves compared to controls at P5 are involved in cell cycle regulation, cell division and DNA replication; this is the reverse of differential gene-set enrichment when compared with that of *TazYap*^dcKO^ nerves (Fig. 7m).

### Gαs signalling antagonizes YAP/TAZ-mediated SC proliferation

**Figure 8:**
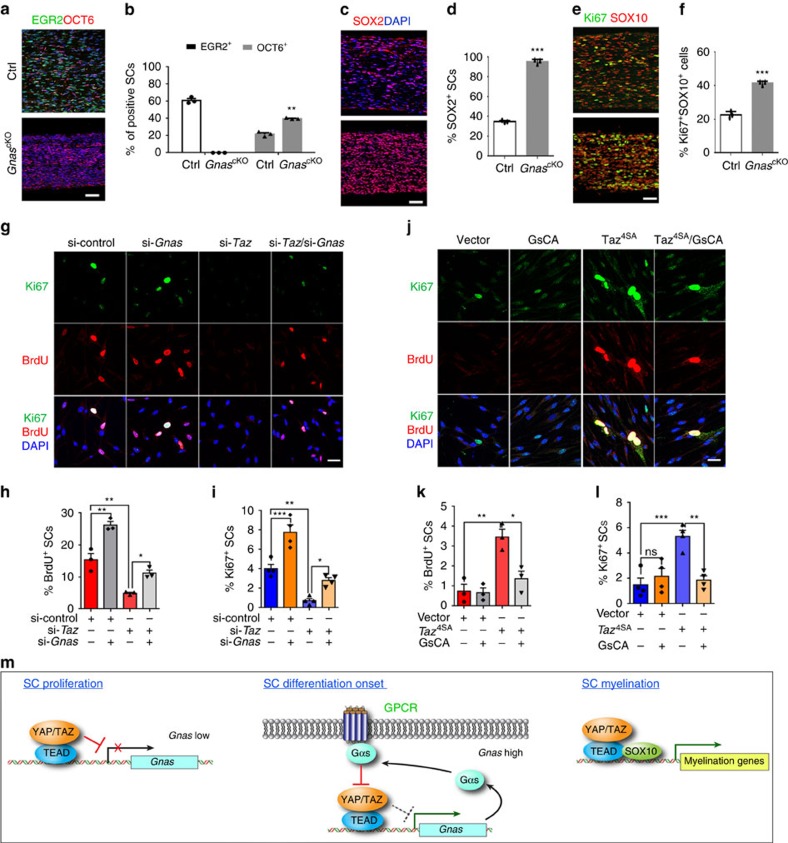
Gαs antagonizes YAP and TAZ to control SC proliferation. (**a**,**b**) EGR2 and OCT6 immunostaining (**a**) and quantification (**b**) in the sciatic nerves of control and *Gnas*^cKO^ sciatic nerves at P7. Nuclei were counterstained with DAPI. *n*=3 animals/genotype. (**c**,**d**) SOX2 immunostaining (**c**) and quantification (**d**) in the sciatic nerves of control and *Gnas*^cKO^ sciatic nerves at P7. *n*=3 animals/genotype. (**e**,**f**) Ki67 and SOX10 immunostaining (**e**) and quantification of Ki67^+^SOX10^+^ cells relative to SOX10^+^ cells (**f**) in the control and *Gnas*^cKO^ sciatic nerves at P7. *n*=3 animals per genotype. The experiment was repeated on 3 different sections per animal. (**g**) Immunofluorescence images show BrdU and Ki67 expression in rat SCs with treated with indicated siRNAs, cultured in neuregulin-supplemented SC growth media. (**h**,**i**) Quantification of (**h**) BrdU^+^ and (**i**) Ki67^+^ cells among SCs transfected with indicated siRNA (*n*=3 (**h**) and 4 (**i**) independent experiments). BrdU pulse was for 6 h. (**j**) Immunofluorescence images show BrdU and Ki67 expression in rat SCs transfected with vectors indicated. Cells were cultured in mitogen-deprived DMEM/10% FBS. BrdU pulse was for 6 h. (**k**,**l**) Quantification of the proportion of (**k**) BrdU^+^ and (**l**) Ki67^+^ SCs transfected with indicated vectors (*n*=3 (**k**) and 4 (**l**) independent experiments). (**m**) Schematic diagram of TAZ/YAP and Gαs-signalling feedback-loop in regulating the transition from SC proliferation to differentiation. Under SC proliferation conditions, YAP/TAZ repress *Gnas* expression and promote SC proliferation. At the onset of SC differentiation, *Gnas* expression is upregulated and Gαs functions as a negative feedback regulator to attenuate TAZ/YAP activity. During myelin formation when Gαs levels decline, YAP and TAZ cooperate with SOX10 to activate the transcriptional programme necessary for SC myelination. Data are presented as means±s.e.m. Each experiment was repeated 3 times. *P<0.05, **P<0.01; ***P<0.001. (**b**,**d**,**f**) Two-tailed unpaired Student's *t-*test; (**h**,**i**,**k**,**l**); one-way ANOVA with Tukey's multiple comparisons test. Scale bars in **a**,**c**,**e**=50 μm; **g**,**j**=25 μm.

In *Gnas*^cKO^ sciatic nerves at P7, we detected an increase in OCT6^+^ immature/pre-myelinating SCs, which failed to mature into EGR2^+^ myelinating SCs (Fig. 8a,b). *Gnas*^cKO^ SCs persistently expressed the immature marker SOX2, suggesting a blockade of SC lineage progression beyond the immature stage (Fig. 8c, d). Notably, whereas control SCs gradually stop proliferating as myelination proceeds, *Gnas*^cKO^ SCs remained robustly proliferative, as revealed by an increase in Ki67^+^ SCs (Fig. 8e,f), resulting in an escalation in immature SC number in the mutant nerves. These data suggest that SCs fail to exit the cell cycle in the absence of *Gnas*.

To further confirm that downregulation of *Gnas* by TAZ promotes SC proliferation, we depleted cultured SCs of *Taz* or *Gnas* or both by siRNA-mediated inhibition in proliferation media without forskolin to prevent upstream Gαs-protein signalling. TAZ deficiency efficiently reduced the numbers of BrdU^+^ proliferative SCs, consistent with its critical role in SC proliferation, while inhibition of *Gnas* expression alone in SCs recapitulated the hyper-proliferation phenotype seen in postnatal *Gnas*^cKO^ peripheral nerves (Fig. 8g–i). Strikingly, inhibition of both *Taz* and *Gnas* in SCs restored proliferation to levels in cells treated with control siRNAs (Fig. 8g–i). Conversely, we performed *Gnas* gain-of-function studies in SCs maintained in mitogen-depleted media. Despite the absence of neuregulin and forskolin, conditions in which SC proliferation was severely limited (Fig. 8j), constitutive TAZ activation forced a population of SCs to proliferate, as indicated by an increase in BrdU^+^ and Ki67^+^ SCs, which were barely detected in control SCs (Fig. 8j–l). Combined expression of activated TAZ and GsCA, a constitutively activated form of Gαs (ref. 44), effectively rescued the over-proliferation defect observed in TAZ-expressing SCs (Fig. 8j–l). Therefore, the balance between TAZ/YAP and Gαs activities is critical for regulating SC proliferation and subsequent maturation.

## Discussion

The opposing cell proliferation and differentiation processes are tightly regulated to ensure proper timing of SC lineage progression. The underlying mechanisms that coordinate these processes are not fully understood. Our studies with SC lineage-specific and developmentally regulated mutagenesis approaches indicate that Hippo signalling effectors TAZ/YAP are required for SC proliferation, radial sorting and differentiation. SC proliferation is a prerequisite for the subsequent events of differentiation and myelination. Activation of TAZ/YAP is sufficient to induce SC proliferation, consistent with genome occupancy analysis showing that TAZ targets cell proliferation-associated genes including canonical Hippo-downstream effectors and cell cycle regulators.

A recent study suggested that dysmyelination in mutants in which *Taz*/*Yap* were deleted using the P0-Cre line are mainly due to radial sorting defects^22^. In the present study, we found that mice with *Taz*/*Yap* deletion in SC precursors using *Dhh-Cre* exhibit severe deficits in SC number and proliferation. The discrepancy could be due to the Cre lines: *Dhh-Cre* is expressed in earlier SC precursors beginning at E12.5 than is P0-Cre, which is expressed around E13.5 (refs 31, 45), raising the possibility that SC proliferation is regulated during the window between the inception of Dhh and P0 promoter activities. Similarly, the lack of substantial radial sorting defect in *Taz*^cKO^ single mutants at P7 (Supplementary Fig. 2) may attribute to *Dhh-Cre*-mediated recombination, although we cannot exclude the possibility that there will be a sorting defect at later postnatal stages as examined in the previous report^22^. Our analysis demonstrated that the TAZ/YAP effect is dosage-dependent since a heterozygous allele in either *Yap* or *Taz* compensates for SC proliferation defects to certain extent. Deletion of *Yap* or *Taz* resulted in compensatory upregulation of expression of the non-deleted *Taz* or *Yap* gene product (Fig. 2c), suggesting that YAP and TAZ are functionally redundant and that their expression levels are tightly regulated to maintain homeostatic state of SC development.

Although myelination-associated laminin receptor genes and *Krox20* are reported as YAP/TAZ targets^22^,^26^, our genome occupancy and transcriptome profiling analyses revealed a global view of TAZ-targeted gene loci including those encoding essential SC differentiation regulators SOX10 and ZEB2 along with neuregulin receptors ERBB2/3 (refs 46, 47), in addition to the reported targets. Our data further indicate that TAZ co-occupies with SOX10 on TEAD-binding sites in SCs, consistent with a recent finding of association of SOX10 binding sites with TEAD in the PNS, but not the CNS^48^,^49^. TAZ/SOX10 co-targeted elements are also associated with the activating H3K27Ac in the gene loci including *Sox10, Zeb2* and *Erbb2/3*, encoding factors that positively regulate SC differentiation and myelination^50^,^51^,^52^,^53^. Surprisingly for what has been hitherto known as a co-activator, TAZ occupancy without SOX10 and H3K27Ac marks appears to repress expression of the genes associated with negative regulation of SC differentiation, suggesting that TAZ functions as a repressor in this context. Furthermore, by using *Plp*-*CreERT*-induced *Taz/Yap* deletion, we showed that *Taz/Yap* are required for initiation of differentiation from immature SCs and myelin formation. Intriguingly, similar to a recent study from Grove *et al*.^26^, deletion of *Taz/Yap* in adult mice by *Plp*-*CreERT* resulted in severe tremors and ataxia. The mice died within 1 month after tamoxifen treatment. In contrast to the Grove et al. study, we did not observe appreciable alteration in myelin thickness or axonal integrity in peripheral nerves after 36 days post tamoxifen treatment (Supplementary Fig. 4). Although the exact mechanism underlying the discrepancy remains to be determined, it is possible that the use of different *Plp*-*CreERT* lines^35^,^54^ or genetic backgrounds may account for different phenotypes in myelin maintenance in sciatic nerves. In addition, TAZ/YAP may regulate physiological properties of SCs and axo-glial interactions, or perhaps, that the essential function of TAZ/YAP in *Plp*-expressing non-SCs such as spleen, liver, kidney and lung^55^ may cause animal death besides demyelination. YAP was reported to play a role in oligodendrocyte morphology and maturation in response to mechanical factors^56^, however, we did not detect overt myelin abnormalities in the adult corpus callosum or spinal cord of *TazYap*^iKO^ mice (Supplementary Fig. 6), suggesting that TAZ/YAP are not required for CNS myelin maintenance. Collectively, our observations suggest that TAZ/YAP are critical for SC proliferation and the myelination onset during SC development.

A recent study indicates that TAZ and YAP are present predominantly in the cytoplasm of SCs at high cell density, and that mechano-stimulation promotes their translocation from the cytoplasm to the nucleus^22^. Intriguingly, in cultured SC and in intact sciatic nerves, we detected expression of TAZ and YAP primarily in the nuclei of SCs at both low and high density, consistent with their function as transcriptional regulators. The underlying discrepancy is likely due to different culture conditions since our finding is consistent with the previous study^22^ when SCs were plated in glass coverslips, indicating that intracellular localization is context-dependent for TAZ and YAP.

Recent studies indicate that Hippo effectors acts downstream of GPCR signalling^19^. Activation of Gαs-coupled GPCRs has been shown to inhibit TAZ/YAP nuclear translocation and their activity^19^,^57^. Consistent with Gαs function, Gpr126, which is able to couple to Gαs, is required for proper SC development and myelination^58^,^59^. Strikingly, we found that Gαs-encoding *Gnas* itself is a direct transcriptional target of TAZ. Despite TAZ being observed thus far to be a co-activator, activation of TAZ/YAP inhibits expression of *Gnas*. Gαs protein levels are low under proliferative conditions and upregulated during SC differentiation. Consistently, *Gnas* deletion leads to excessive SC proliferation while inhibiting SC differentiation, suggesting that Gαs protein levels regulate the transition of SC proliferation to differentiation. Radial sorting defects in *Gnas* mutants appear less severe compared to the *Taz/Yap* double mutant^26^,^58^ (Supplementary Figs 2 and 5) or *Gpr126* mutant^22^,^26^,^58^, this suggests that these pathways may regulate radial sorting via additional mechanisms other than Gαs-dependent mechanisms. Nonetheless, our findings identify a crucial role of the reciprocal signalling of TAZ/YAP and Gαs in regulating SC proliferation-to-differentiation transition. *Gnas* knockdown in SCs lead to a downregulation of cAMP levels (Supplementary Fig. 7). Intriguingly, Oct6 appears upregulated in *Gnas* and *Taz/Yap* mutant sciatic nerves^26^. Since Oct6 is a downstream target of cAMP signalling in SCs, it is possible that Gαs functions in cAMP-dependent and -independent mechanisms during SC development in sciatic nerves, or perhaps, other pathways may activate Oct6 expression in the absence of Gαs-signalling in *Gnas*-mutant nerves.

Given that YAP/TAZ remain active in adult SCs, Gαs downregulation at late developmental stages in differentiated SCs may allow the pro-differentiation effects of TAZ/YAP, in collaboration with SOX10, on regulation of the SC myelination programme to prevail (Fig. 8m). This suggests that changes in G-protein levels may account for a switch in the net effect of YAP/TAZ on proliferation versus differentiation during SC development in a stage-dependent manner.

Our ChIP-seq data indicate that TAZ targets an intronic region in the *Gnas* locus. Although transcriptional regulators can bind to regions of open chromatin without functional effect^60^. ChIP-PCR confirms that TAZ binds to the targeted sites on the *Gnas* locus. By overexpression of TAZ or YAP, *Gnas* transcription is attenuated, while reduction of TAZ/YAP by siRNA knockdown leads to an upregulation of *Gnas,* suggesting that TAZ may transcriptionally represses *Gnas* expression. It is worth noting that since the expression pattern of *Gnas* does not strictly correlate with that of *Taz* and *Yap* mRNA, it is unlikely that TAZ is the sole repressor of *Gnas* expression, which could be also regulated by other mechanisms. Conversely, *Gnas* deficiency leads to an upregulation of *Taz*/*Yap* as well as *Gli1-3* expression in *Gnas*-mutant nerves (Supplementary Fig. 8). This is consistent with Gαs function as a tumour suppressor that inhibits Sonic hedgehog signalling and Hippo signalling-mediated cell proliferation in medulloblastoma or basal-cell carcinoma^44^,^61^,^62^, suggesting a reciprocal and antagonistic function between Gαs signalling and Hippo/hedgehog pathways.

Our findings place Gαs-mediated signalling in an unanticipated role as a key modulator that antagonizes TAZ/YAP effects during the onset of SC differentiation. We propose that the TAZ/YAP/Gαs regulatory loop acts as a feedback circuit that balances SC proliferation and differentiation (Fig. 8m). As constitutive activation of either YAP or TAZ enhances proliferation of cultured SCs, this Gαs regulatory loop may serve to limit SC proliferation. By contrast, Gαs activation results in reduced SC proliferation, whereas *Gnas* deletion leads to a drastic increase in SC proliferation, consistent with a tumour suppressor function of *Gnas* in other contexts^44^,^61^. Thus, our findings establish Gαs as a critical node in the inhibitory feedback-loop that antagonizes TAZ/YAP activity in regulating the balance between SC proliferation and differentiation. Future studies aimed at modulating or fine-tuning the dynamic balance between YAP/TAZ and Gαs may lead to therapeutic strategies to promote myelination in the PNS while inhibiting SC over-proliferation that occurs during formation of peripheral nerve sheath tumours such as neurofibromas and Schwannomas.

## Methods

### Animals

Mice homozygous for floxed alleles of *Taz* (*Taz*^*lox*/*lox*^); *Yap* (*Yap*^*lox*/*lox*^)^30^ and *Gnas*^*lox/lox*^ mice^63^ were crossed with mice carrying *Dhh-Cre*^31^ to generate *Taz* and Yap single and double mutant mice or *Gnas*^*cKO*^ mice, respectively. Inducible knockouts, iKO, for recombination perinatally or following sciatic nerve transection were generated by crossing *Taz*^*lox/lox*^*;Yap*^*lox/lox*^ mice with the inducible Cre recombinase Cre-ERT2 under the control of the *Plp* promoter (*Plp-*Cre-ERT)^35^ followed by tamoxifen injection. *Taz*^*lox/lox*^*;Yap*^*lox/lox*^*; Plp-Cre-ERT* mice were also bred to ccGFP reporter mice^36^. Animals of either sex were used in the study, and littermates were used as controls unless otherwise indicated. The mouse strains used in this study were generated and maintained on a mixed C57Bl/6;129Sv background and housed in a vivarium with a 12-hour light/dark cycle. No more than 4 adult mice are housed in the same cage per IACUC regulations at CCHMC. All animal use and studies were approved by the Institutional Animal Care and Use Committee at Cincinnati Children's Hospital, USA.

### Tamoxifen induction of gene deletion

Tamoxifen (Sigma) was dissolved to a stock concentration of 20 mg ml^−1^ in a vehicle of ethanol and sunflower seed oil (1:9 v/v). For perinatal tamoxifen injections, tamoxifen stock was injected i.p. at 2 mg per 100 μl for 10 consecutive days to lactating mothers, thus administering tamoxifen to pups, beginning at P0. Sciatic nerves of pups were analysed on P14 for immunofluorescence staining and electron microscopy. For adult tamoxifen treatment, 100 μl (2 mg per 100 μl) was administered by i.p. injection once daily for 5 consecutive days to 6–8 week-old mice. Mice were treated again for 5 days after a 1-day rest period. Control mice were treated identically.

### Primary Rat SC culture

Rat SCs from sciatic nerves of newborn rats (1–2 days-old) were isolated as described previously^64^. SCs were grown routinely in DMEM/10% fetal bovine serum (FBS) (Life Technologies), supplemented with 10 ng ml^−1^ Neuregulin 1 (R&D Systems), and 5 μM forskolin (Sigma), plus L-glutamine and penicillin/streptomycin, hereafter denoted as SC growth/proliferation medium. Cells between passages 2 and 6 were used in all experiments. More than 95% SC purity was achieved, assessed by positive SOX10 and S100β immunoreactivity. To initiate differentiation, SCs were cultured in differentiation medium containing DMEM/0.5% FBS and 1 mM dibutyl cyclic AMP (Sigma) with L-glutamine and penicillin/streptomycin, for the length of time indicated in the text, depending on the assays used. All tissue culture containers and glass coverslips (Carolina Biological cat# 633029) were coated with 50 μg ml^−1^ poly-L-lysine (Sigma) in phosphate buffered saline (PBS) for at least 30 min at room temperature and then rinsed with distilled water. For BrdU incorporation analysis, BrdU at 20 μM was added to purified rat SCs seeded on coverslips 24 h or otherwise indicated, prior to fixation in 4% (w/v) paraformaldehyde (PFA).

### Immunofluorescence staining

The sciatic nerves of mice at defined ages were dissected and fixed for 15 min in 4% PFA in 0.1 M sodium phosphate buffer (pH 7.4), embedded in OCT, cryoprotected in 25% sucrose and sectioned at 12 μm as longitudinal or cross-sections using a cryostat or at 30 μm using a vibratome. Teased fibres were prepared by separating and teasing nerve bundles into individual fibres with acupuncture needles in a drop of PBS (pH 7.4) on glass slides followed by air-drying.

For BrdU pulse labelling, animals were injected subcutaneously or intraperitoneally with 100 mg BrdU kg^−1^ body weight 2 h prior to sciatic nerve collection. Tissue sections or cells were permeabilized and blocked in blocking buffer (0.3% Triton X-100 and 5% normal donkey serum in PBS) for 1 h at 25 °C, followed by incubating with primary antibodies overnight at 4 °C. We used antibodies to TAZ (Rabbit; Cell Signaling Technology, #4883, dilution 1:200), YAP (Rabbit; Cell Signaling Technology, #4912, dilution 1:200), YAP/TAZ (Cell Signaling Technology, #8418S, dilution 1:200), SOX10 (Goat; Santa Cruz Biotechnology, sc-17342, dilution 1:200), OCT6 (Goat; Santa Cruz Biotechnology, sc-11661, dilution 1:200), EGR2/KROX20 (Rabbit; Santa Cruz, sc-20690, dilution 1:200), MBP (Goat; Santa Cruz Biotechnology, sc-13914, dilution 1:500), MAG (Millipore, MAB1567, dilution 1:500), SOX2 (Goat; Santa Cruz Biotechnology, sc-17320, dilution 1:200), Ki67 (Rabbit; Thermo Scientific, RM-9106, dilution 1:500), BrdU (Rat; Abcam, ab6326, dilution 1:500), Cleaved Caspase 3 (Rabbit; Cell Signaling Technology, #9661, dilution 1:500), S100β (Mouse; Sigma, SAB1402349, dilution 1:1,000). Secondary antibodies conjugated to Cy2, Cy3 or Cy5 were from Jackson ImmunoResearch Laboratories (dilution of secondary antibodies is 1:1,000). All images were acquired using a Nikon C2^+^ confocal microscope.

### Electron microscopy and morphometric analysis

Mice were perfused with 4% PFA, 2.5% glutaraldehyde in 0.1 M sodium cacodylate buffer, pH 7.2. Sciatic nerves were dissected and fixed in the same fixative solution overnight. Nerves were rinsed in PBS, postfixed in 1% OsO4 in PBS for 1 h, dehydrated in graded ethanol, infiltrated with propylene oxide, and embedded in Epon. Semithin sections were stained with toluidine blue, and ultrathin sections were stained with lead citrate. The morphometric measurements of axonal sorting defects were performed using electron micrographs of ultrathin sections and analysed using NIH Image J software (http://rsb.info.nih.gov/ij/). An entire sciatic nerve cross-section per animal was reconstructed by merging several high magnification photographs taken by bright field microscopy at 100 × magnification. The number of myelinated axons per nerve were analysed in ultrathin sections using a JEOL 1200 EXII electron microscope.

### Transient transfections and luciferase assays

For plasmid transfections, HEK293 cells in DMEM/10% FBS were transfected with expression vectors using Polyjet (SignaGen, SL100688) per the manufacturer's protocol and assayed for immunocytochemistry or quantitative polymerase chain reaction with reverse transcription (qRT-PCR) analysis.

For siRNA knockdown in SCs, we used Lipofectamine RNAiMAX (Life Technologies, cat# 1377850) per manufacturer's instructions. SCs were plated in SC proliferation medium. Prior to siRNA knockdown, SC medium was changed to DMEM/10% FBS with 10 ng ml^−1^ Neuregulin 1 only. SCs were transfected with scrambled siRNA control or specific siRNAs against *Taz*, *Yap* or *Gnas* for 48 h before being subject to immunocytochemistry or qRT-PCR analysis. For double siRNA knockdown, two gene specific siRNAs were prepared in the same transfection mix before adding to the cells. siRNAs were purchased from Sigma-Aldrich with the following catalogue numbers: for rat *Gnas*, SASI_Rn02_00202731 and SASI_Rn02_00202732; for rat *Taz,* SASI_Rn02_00202747 and SASI_Rn01_00120445; for rat *Yap*, SASI_Rn01_00114056 and SASI_Rn01_00114054.

Proliferation was assessed by immunostaining for Ki67 and BrdU in SOX10^+^ SCs seeded on poly-L-lysine (PLL)-coated coverslips. Differentiation was assessed by immunostaining for OCT6 or EGR2. Multiple images were taken from each coverslip to obtain representative images from all areas of the coverslip, and at least 400 cells/coverslip were counted using Nikon NIS-Elements software.

For reporter assays, HEK293 cells were transfected with pGL3-luciferase reporters driven by indicated promoters to TSS sites (*Mbp*, −1,323 bp to +3 bp; *Mpz*, −1,100 kb to + 0 bp; *Hes1*, −467 bp to +46 bp; *Hes5*; −800 bp to +73 bp; *c-Jun*, −225 bp to +150 bp) using Polyjet per the manufacturer's protocol and assayed 48 h, post-transfection for luciferase activity by using a Promega luciferase assay kit according to the manufacturer's instructions. The pSV-β-galactosidase control vector was included to control for variable transfection efficiencies between independent experiments.

### Western blotting

For western blotting, the perineurium and epineurium were removed from sciatic nerves prior to snap-freezing and storage at −80 °C. Sciatic nerves and rat SCs were lysed in RIPA buffer, containing protease and phosphatase inhibitors. Western blot analysis was performed as described previously^52^. Gapdh (Millipore MAB374, dilution 1:1,000) was used as an input control. The antibodies used were anti-TAZ (Novus biologicals, NB11058359, dilution 1:1,000), YAP (Rabbit; Cell Signaling Technology, #4912, dilution 1:1,000), anti-p-YAP (Cell Signaling Technology, #13008S, dilution 1:1,000) anti-EGR2/KROX20 (Rabbit; Santa Cruz, sc-20690, dilution 1:1,000) and Anti-Gαs (Rabbit; Santa Cruz sc-823, dilution 1:1,000). Secondary antibodies conjugated to HRP were from Jackson ImmunoResearch Laboratories (dilution of secondary antibodies is 1:10,000). Full-size images for the main figures and Supplementary Figs are presented in Supplementary Fig. 9.

### RNA isolation and quantitative real-time PCR

RNA from purified rat SCs or mouse sciatic nerves was extracted using TRIZOL (Life Technologies). cDNA was synthesized from RNAs using iScript Reverse Transcription Supermix (BioRad) according to the manufacturer's instructions. qRT-PCR was performed using the StepOnePlus Real-time PCR System (Applied Biosystems). qRT-PCR was performed using quantitative SYBR green PCR mix (BioRad). Comparative ΔΔCT were performed and normalized to *Gapdh* as an internal control. PCR primer sequences are available in Supplementary Table 2.

### Enzyme-linked immunoassay for cAMP

Rat SCs were plated on 12-well plates and cultured for 1 day. Cells were transfected with control and *Gnas* siRNA and cultured in the SC growth medium without forskolin overnight. The cAMP level in SC lysates was assayed following the manufacturer's protocol (Cell Signaling, cat# 4339).

### RNA-sequencing and data analysis

RNA from control, *Taz/Yap* mutant sciatic nerves with epineurium removal were extracted using TRIZOL (Life Technologies) followed by purification using an RNeasy Mini Kit (Qiagen). To minimize the potential impact of a reduced total number of SCs in the dissected sample, we used a similar amount of total RNAs from control and mutant sciatic nerves was used for RNA-seq analysis. RNA-seq libraries were prepared using Illumina RNA-Seq Preparation Kit and sequenced by HiSeq 2000 sequencer. RNA-seq reads were aligned to mm10 using TopHat with default settings (http://tophat.cbcb.umd.edu). We used Cuff-diff to (1) estimate fragments per kilobase of transcript per million (FPKM) values for known transcripts and (2) analyse differentially expressed transcripts. In all differential expression tests, a difference was considered significant if the q value was less than 0.05 (Cuff-diff default). Heatmap of gene expression was generated using R language and was generated based on log2 (FPKM).(http://www.r-project.org). GO analysis of gene expression changes was performed using Gene Set Enrichment (GSEA, http://www.broadinstitute.org/gsea/index.jsp). We used ToppCluster (https://toppcluster.cchmc.org/) to construct the network of genes belonging to over-represented GO-term categories.

### Chromatin immunoprecipitation sequencing and ChIP-qPCR assay

ChIP assays were performed as described previously with minor modifications^52^,^65^. Briefly, purified rat SCs (∼20 million cells) were fixed for 10 min at room temperature with 1% formaldehyde-containing medium. Nuclei were isolated and sonicated in sonication buffer (10 mM Tris-HCl [pH 8.0], 1 mM EDTA, 0.5 mM EGTA and protease inhibitor cocktail). Sonicated chromatin (∼300 μg) was used for immunoprecipitation by incubation with Rabbit IgG (Santa Cruz, SC-2027, 4 μg) or anti-Taz antibody (Sigma, HPA007415, 4 μg), or anti-H3K27Ac (Abcam, ab4729, 4 μg) overnight at 4 °C. 10% of chromatin used for each ChIP reaction was kept as input DNA. Pre-rinsed protein A/G plus agarose beads (50 μl) was added to each ChIP reaction and incubated for 1 h at 4 °C. The beads were then incubated in 200 μl elution buffer at 65 °C for 20 min to elute immunoprecipitated materials. Real-time PCR was performed using quantitative SYBR green PCR mix (BioRad). The relative fold-enrichments were determined by the 2^−ΔCT^ methods. Samples were normalized to input chromatin. The ChIP-seq libraries were prepared using NEBNext ChIP-seq Library Prep Master Mix Set for Illumina (NEB catalogue number E6240L) and then run on the Illumina sequencer HiSeq 2000. Primers used for ChIP-qPCR analysis: *Gnas*: Forward 5′-ACTGGAATTCAGCCTCTGTG-3′; Reverse 5′-CAGCGTAGGAATGCGATGTA-3′; *Gnai1-1*: Forward 5′-AGCCATGTTTGGTGGTACAT-3′; Reverse GTCTGGATGGGAATTTGTTGTG-3′; *Gnai1-2*: Forward 5′-GACCAGGCTTCCAAATATCTCC-3′; Reverse 5′-CCTCACAACCA AGTGATGTGTA-3′.

### ChIP-seq peak-calling and data analysis

All sequencing data were mapped to rat genome assembly rn5 and peak calling was performed using model-based analysis of ChIP-seq version 1.4.2 (http://liulab.dfci.harvard.edu/MACS) with default parameters, to get primary binding regions. The ChIP-seq were done in two biological replicates. The numbers of reads per replicate were ∼20–21 million for TAZ ChIP-seq, 19–20 million for H3K27Ac ChIP-seq, respectively. To ensure that our data were of high quality and reproducibility, we called peaks with enrichment ≥10-fold over control (*P*≤10^−9^) and compared the peak sets using the ENCODE overlap rules. These identified primary regions were further filtered using the following criteria, to define a more stringent protein–DNA interactome: (1) the *P* value cut-off was set to ≤10^−9^; (2) an enrichment of 6.8-fold and peak height >5.

The genome-wide distribution of protein binding regions was determined by HOMER (http://homer.salk.edu/homer/index.html) in reference to Ensembl RGSC3.4.61 release. Motif enrichment analysis was performed using Homer software. For all ChIP-seq data sets, WIG files were generated with model-based analysis of ChIP-seq, which were subsequently visualized using Mochiview v1.46. Taz and H3K27Ac ChIP-seq heatmaps were ordered by strength of binding. The heatmaps were drawn using the Heatmap tools provided by Cistrome (http://cistrome.org/ap).

### Statistical analysis

All analyses were done using Microsoft Excel or GraphPad Prism 6.00 (San Diego, California, www.graphpad.com). Data are shown in dot plots or bar graphs as mean±s.e.m., *P*<0.05 is deemed statistically significant. Data distribution was assumed to be normal, but this was not formally tested. Count data was assumed to be non-parametric, and appropriate statistical tests were used. Statistical analysis was performed by two-tailed unpaired Student's *t*-tests, Mann–Whitney test, one-way analysis of variance (ANOVA) with *post hoc* analysis by Tukey's multiple-comparison test, or as indicated. Quantifications were performed from at least three experimental groups in a blinded fashion. No statistical methods were used to predetermine sample sizes, but our sample sizes are similar to those generally employed in the field. No randomization was used to collect all the data, but they were quantified blindly. No animals or data points were excluded from analyses.

### Data availability

All the RNA-seq and ChIP-seq data have been deposited in the NCBI Gene Expression Omnibus (GEO) under accession number GSE94990.

## Additional information

**How to cite this article:** Deng, Y. *et al*. A reciprocal regulatory loop between TAZ/YAP and G-protein Gαs regulates Schwann cell proliferation and myelination. *Nat. Commun.* **8,** 15161 doi: 10.1038/ncomms15161 (2017).

**Publisher's note:** Springer Nature remains neutral with regard to jurisdictional claims in published maps and institutional affiliations.

## Supplementary Material

Supplementary Information Supplementary Figures and Supplementary Tables.
