# Supplementary material for: A reciprocal regulatory loop between TAZ/YAP and G-protein Gαs regulates Schwann cell proliferation and myelination
Source: Nat Commun. 2017 Apr 26;8:15161. doi: 10.1038/ncomms15161 (PMC5414202; doi:10.1038/ncomms15161)
Supplement: Supplementary Information — Supplementary Figures and Supplementary Tables. [file ncomms15161-s1.pdf]

SUPPLEMENTARY INFORMATION

*Deng et al.*

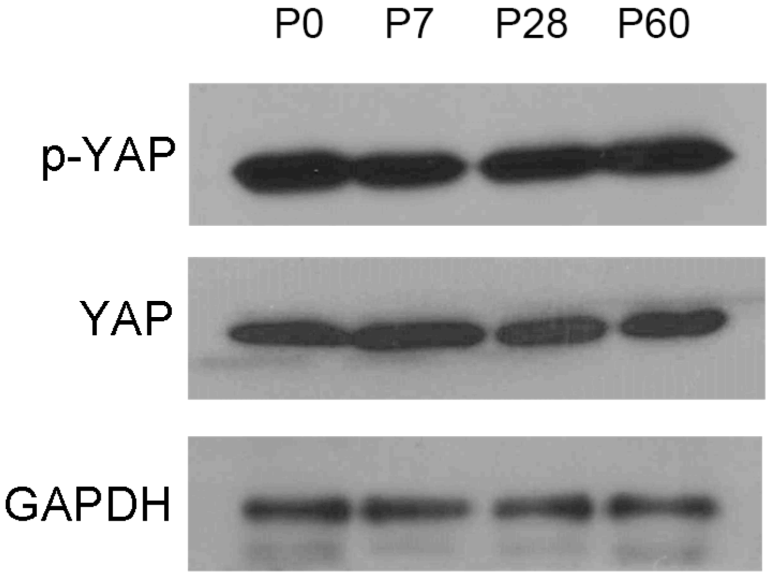


**Supplementary Fig. 1. Phospho-YAP expression levels in developing sciatic nerves**

Representative western blot image of p-YAP and YAP expression in wild type mouse sciatic nerves at indicated stages. GAPDH were used as a loading control. The experiment was Repeated twice on 2 animals at each stages)

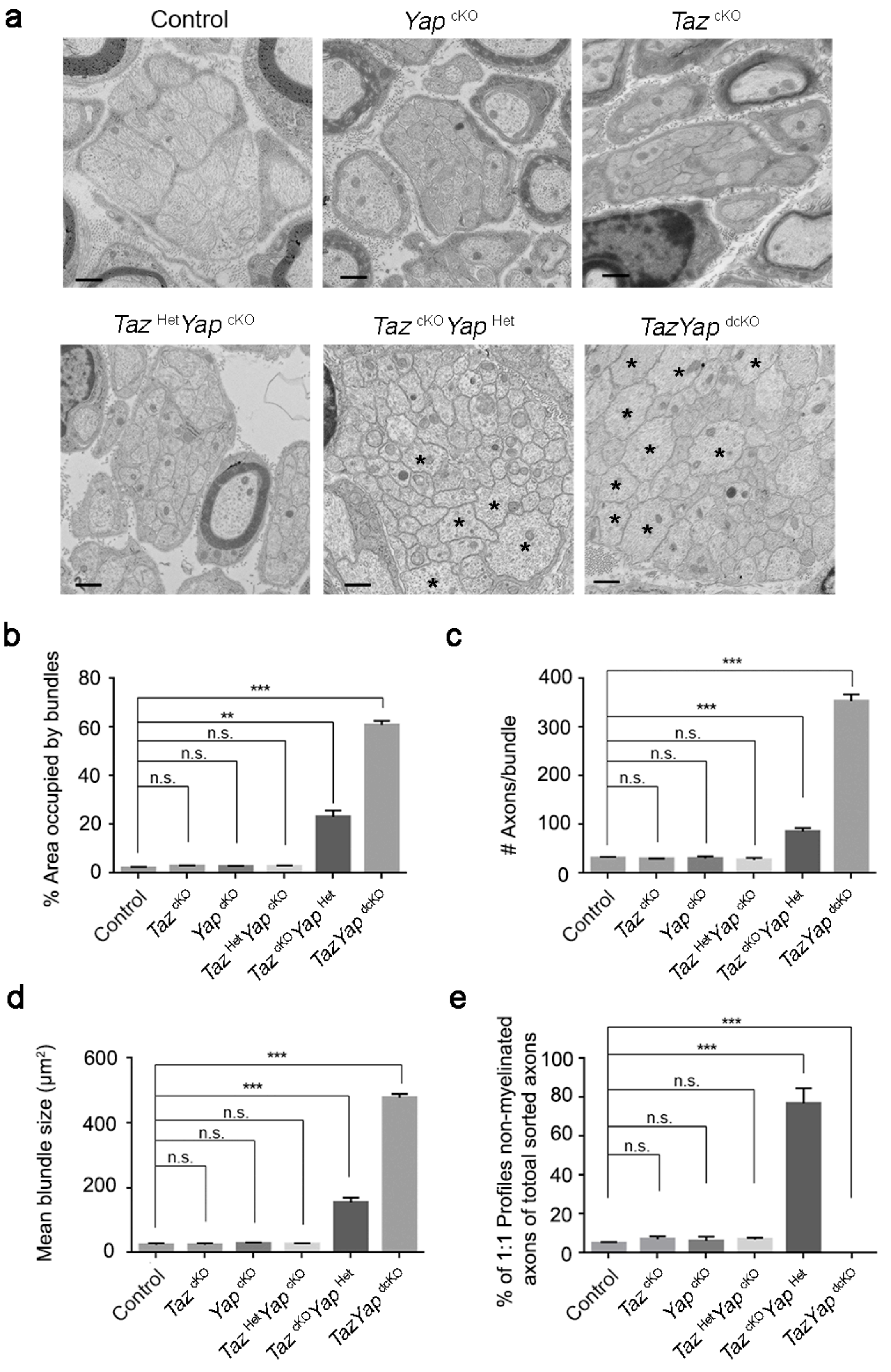


**Supplementary Fig. 2. Radial sorting defect in *TazYap* double mutant sciatic nerves**

**a**) Electron microscopy images showing the unmyelinated axon bundles in control, *Yap*$^{cKO}$, *Taz*$^{cKO}$, *Taz*$^{Het}$*Yap*$^{cKO}$, *Taz*$^{cKO}$*Yap*$^{Het}$, and *TazYap*$^{dcKO}$ sciatic nerves at P7. Scale bar, 1 μm.

**b-e**) Quantification of radial sorting defects in control, *Yap*$^{cKO}$, *Taz*$^{cKO}$, *Taz*$^{Het}$*Yap*$^{cKO}$, *Taz*$^{cKO}$*Yap*$^{Het}$, and *TazYap*$^{dcKO}$ sciatic nerves at P7. Panel **b** shows the percentage of area occupied by naked axon bundles (bundles with unsorted large axons with diameter > 1 μm); panel **c** shows the number of axons per bundle; panel **d** shows the bundle size, and panel **e** shows the percentage of 1:1 profile non-myelinated (naked) axons of total sorted axons. (n = 5 animals/genotype; at least 20 fields are counted in each animal). Data are means ± s.e.m. **p < 0.01, ***p < 0.001; n. s. represents non-significant, one-way ANOVA with Tukey's multiple-comparison test.

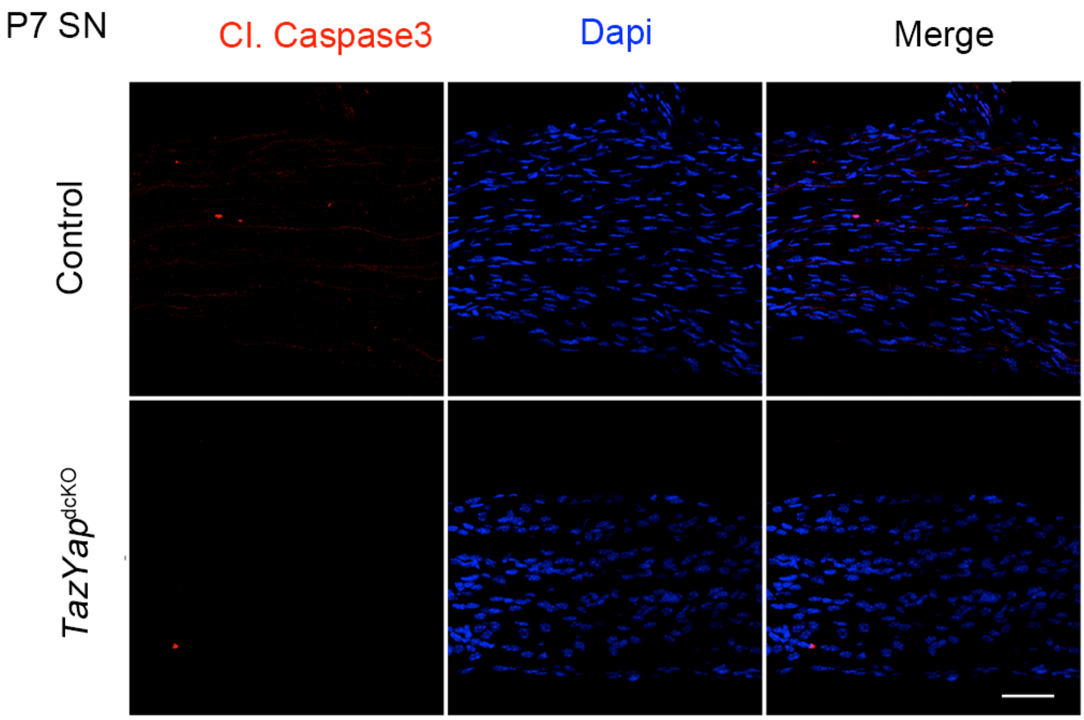


**Supplementary Fig. 3. Absence of significant cell death in developing sciatic nerves of *TazYap* mutants**

Immunofluorescence labeling for cleaved Caspase 3 (red) in control and *TazYap*$^{dCKO}$ mutant sciatic nerves at P7 (n = 3 animals/genotype). DAPI nuclear counterstain is shown in blue. Scale bar, 50 µm.

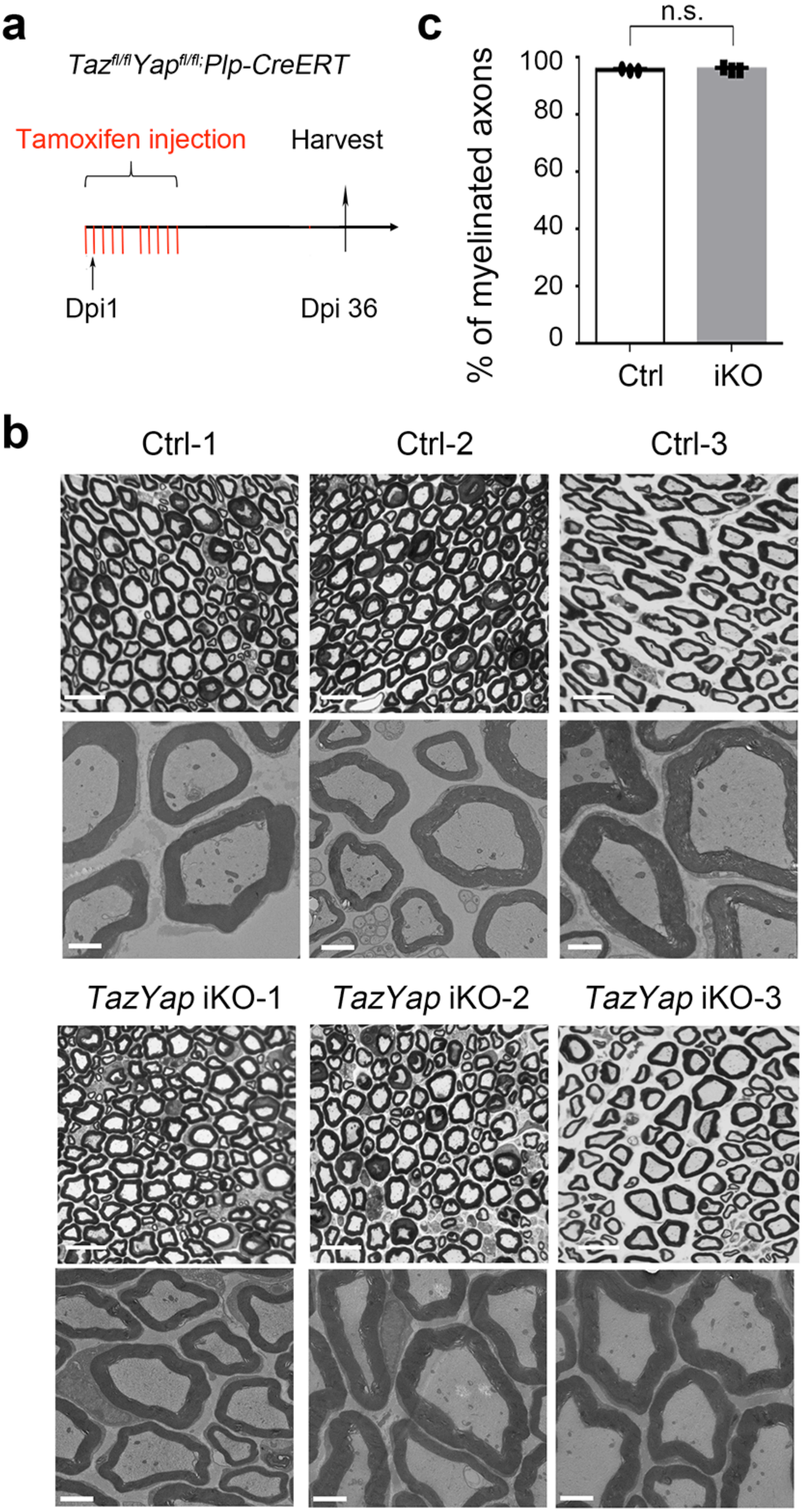
a
Taz^fl/fl Yap^fl/fl;Plp-CreERT
Tamoxifen injection
Harvest
Dpi1
Dpi 36
c
n.s.
% of myelinated axons
100
80
60
40
20
0
Ctrl
iKO
b
Ctrl-1
Ctrl-2
Ctrl-3
TazYap iKO-1
TazYap iKO-2
TazYap iKO-3

**Supplementary Fig. 4. Normal appearance of myelinated axons in the sciatic nerves of *Taz/Yap* inducible knockout adult mice**

**a**) Schematic diagram showing that tamoxifen injection of control (*Plp-CreERT*) and *TazYap*$^{iKO}$ (*Taz*$^{fl/fl}$*Yap*$^{fl/fl}$;*Plp-CreERT*) for 2 five-consecutive days in 7 week-old animals. Sciatic nerves were collected for thick section analysis 36 days post injection (Dpi).

**b**) Representative semithin (upper panel) images and ultra-thin electron microscopy (lower panel) images showing the control and *TazYap*$^{iKO}$ adult sciatic nerves from three individual animals at Dpi 36. All these tamoxifen-treated *TazYap*$^{iKO}$ mice developed severe tremors and ataxia. Scale bar, semithin images: 20 μm, ultra-thin images: 3 μm.

**c**) Quantification the percentage of myelinated axons in control and *TazYap*$^{iKO}$ adult sciatic nerves at Dpi 36 (n = 3 animals/genotype; at least 20 fields are counted in each animal). n. s. represents non-significant. Two-tailed unpaired Student's *t*-test.

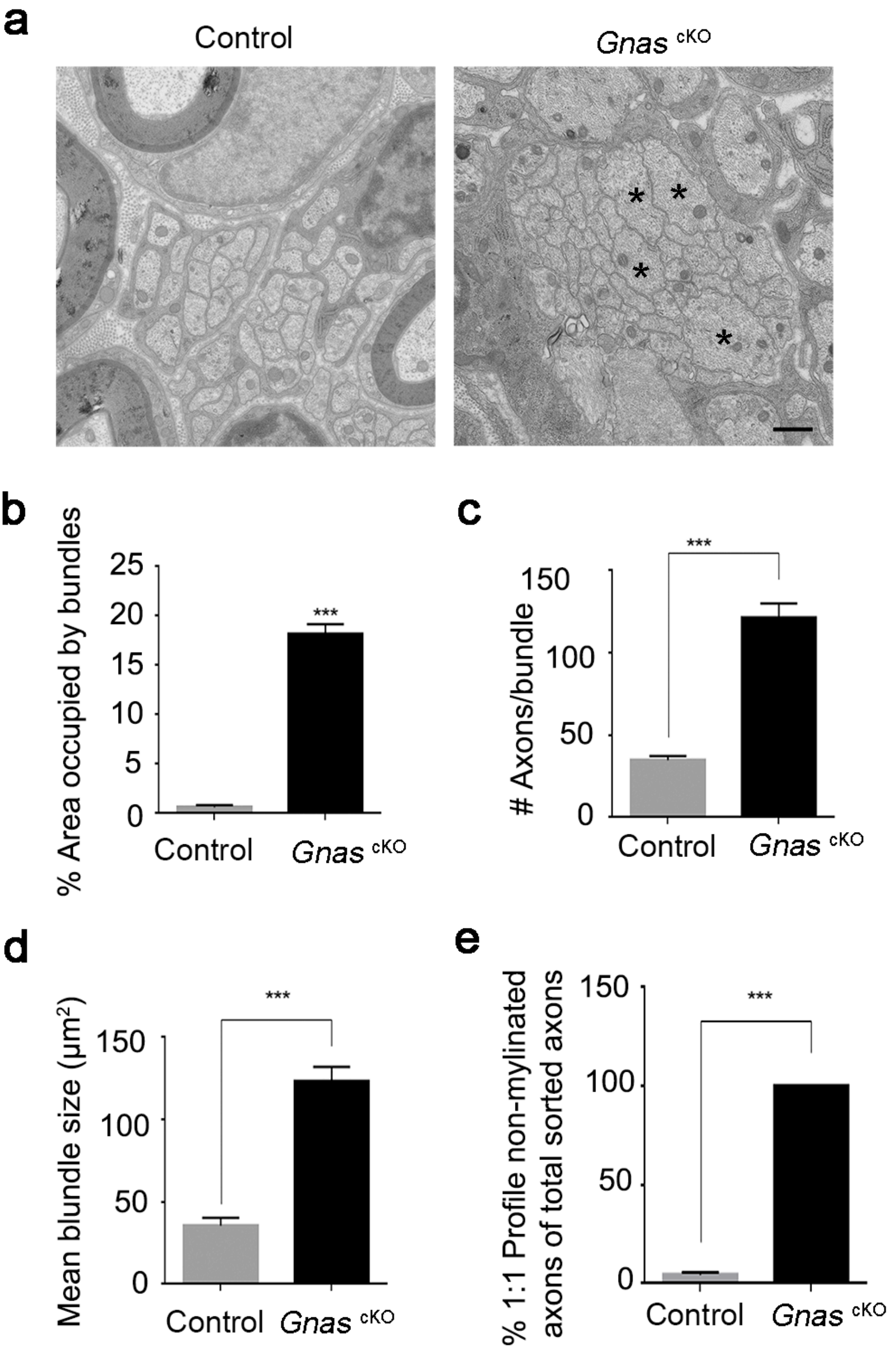


**Supplementary Fig. 5. Radial sorting defect in *Gnas* mutant sciatic nerves**

**a**) Electron microscopy ultra-thin section images of unmyelinated axon bundles in control and *Gnas*$^{cKO}$ sciatic nerves at P10. Scale bar, 1 µm.

**b-e**) Quantification of radial sorting defects in control, and *Gnas*$^{cKO}$ sciatic nerves at P10. Panel **b** shows the percentage of area occupied by naked axon bundles (bundles with unsorted large axons with diameter > 1 μm); panel **c** shows the number of axons per bundle; panel **d** shows the bundle size, and panel **e** shows the percentage of 1:1 profile non-myelinated (naked) axons of total sorted axons. (n = 5 animals/genotype; at least 20 fields are counted in each animal). Data are means ± s.e.m. ***p < 0.001; n. s. represents non-significant. ANOVA with Tukey's multiple-comparison test.

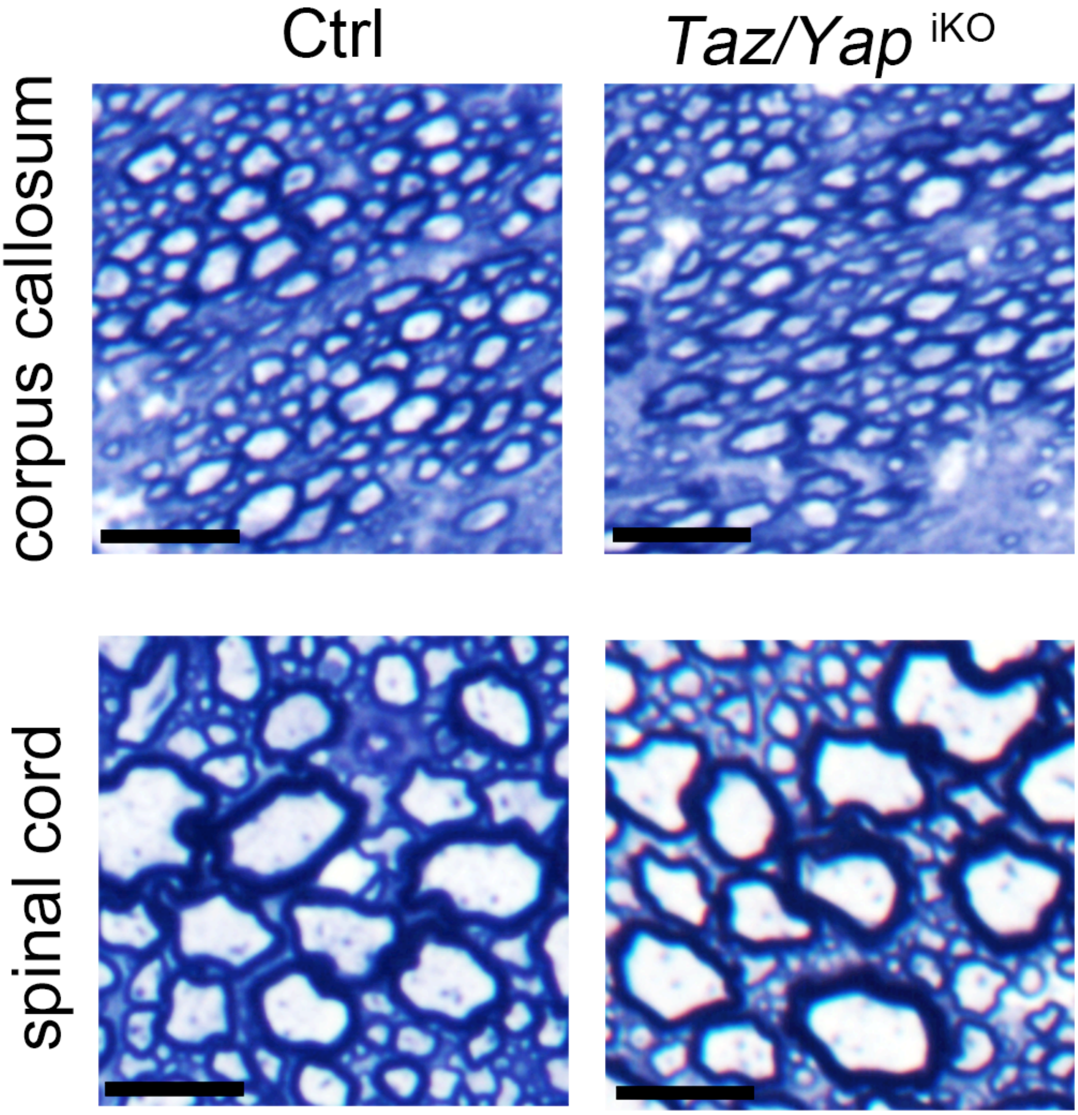


**Supplementary Fig. 6. Normal appearance of myelination in the corpus callosum and spinal cord in *Taz/Yap* inducible knockout adult mice**

Representative semithin section images of the corpus callosum and spinal cord from the wildtype control and *TazYap*$^{iKO}$ adult mice at Dpi 36 (n = 3 animals/genotype). Scale bar, *10* µm.

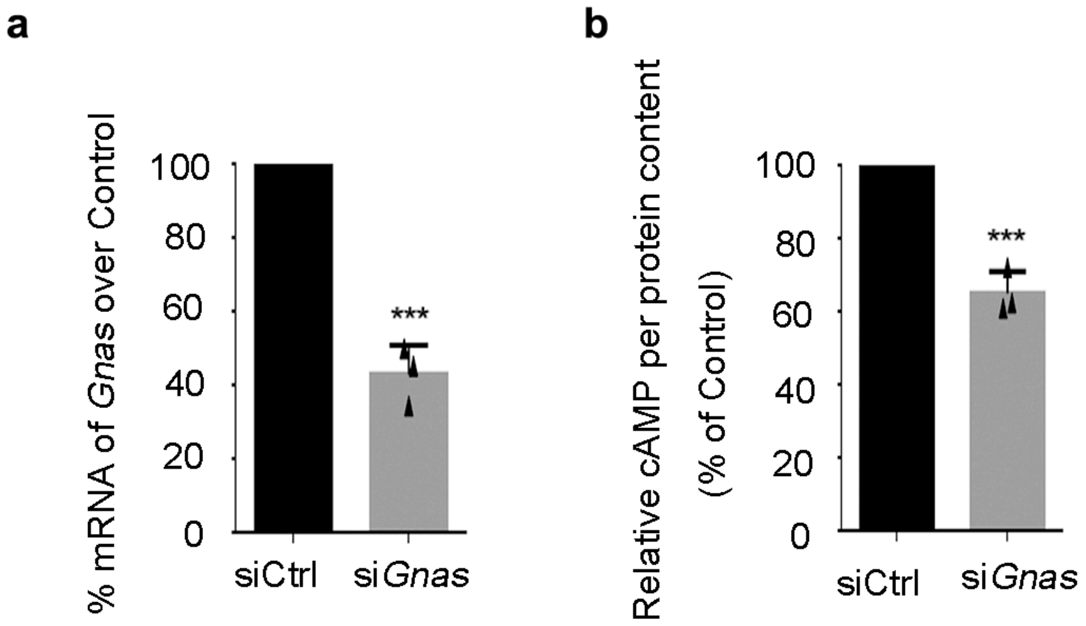


**Supplementary Fig. 7. cAMP assay of control and *Gnas*-knockdown rat Schwann cells**

**a)** qRT-PCR analysis of *Gnas* following treatments with scrambled siRNA control or siRNAs targeted *Gnas* in rat Schwann cells. Data are presented as means ± S.E.M; n = 3 independent experiments. *** $p < 0.001$; Two-tailed unpaired Student's t-test.

**b)** cAMP assay in following treatments with scrambled siRNA control or siRNAs targeted *Gnas* in rat Schwann cells. The cAMP level per protein content of control was normalized as 100%, n = 3 independent experiments, Data are presented as means ± S.E.M; *** $p < 0.001$; Two-tailed unpaired Student's t-test.

**a**

| Genes | Ctrl (FPKM) | *Gnas*$^{cKO}$ (FPKM) | Fold change (*Gnas*$^{cKO}$/$_{Ctrl}$) |
|---|---|---|---|
| *Wwtr1(Taz)* | 36.1903 | 55.0518 | 1.52 |
| *Yap1* | 20.3348 | 25.8748 | 1.27 |
| *Gli1* | 9.12482 | 5.247106 | 5.25 |
| *Gli2* | 0.691816 | 3.75641 | 3.76 |
| *Gli3* | 1.27924 | 4.77242 | 4.77 |

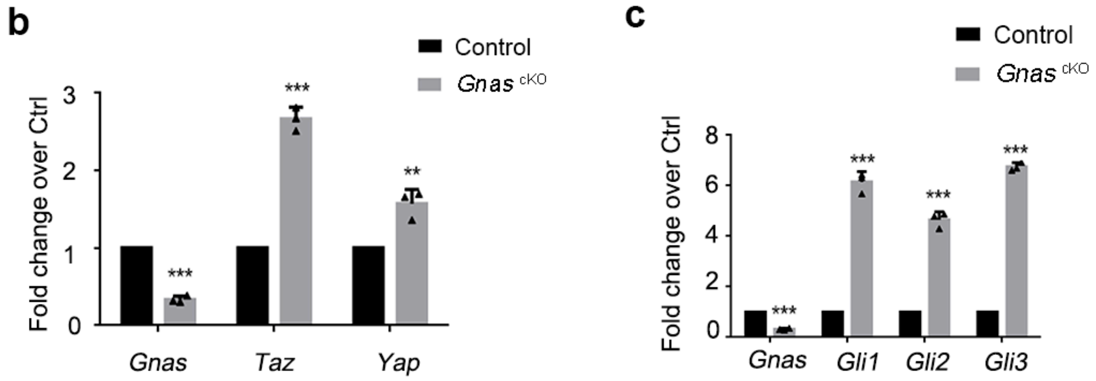


**Supplementary Fig. 8. Transcriptional level changes of *Taz/Yap* and *Glis* in *Gnas*$^{cKO}$ sciatic nerves**

**a**) FPKM values and fold change (cKO/Ctrl) of *Taz* (*Wwtr1*), *Yap,* and *Gli1-3* from RNA-seq of control and *Gnas*$^{cKO}$ mutant sciatic nerves at P5.

**b**) qRT-PCR analysis of *Taz/Yap* with sciatic nerves of control and *Gnas*$^{cKO}$ mutant mice at P7. Data are presented as means ± S.E.M; n = 3 animals/genotype, **p < 0.01, *** p < 0.001; Two-tailed unpaired Student's t-test.

**c**) qRT-PCR analysis of *Gli1-3* with sciatic nerves of control and *Gnas*$^{cKO}$ mutant mice at P7. Data are presented as means ± S.E.M; n = 3 animals/genotype, *** p < 0.001; Two-tailed unpaired Student's t-test.

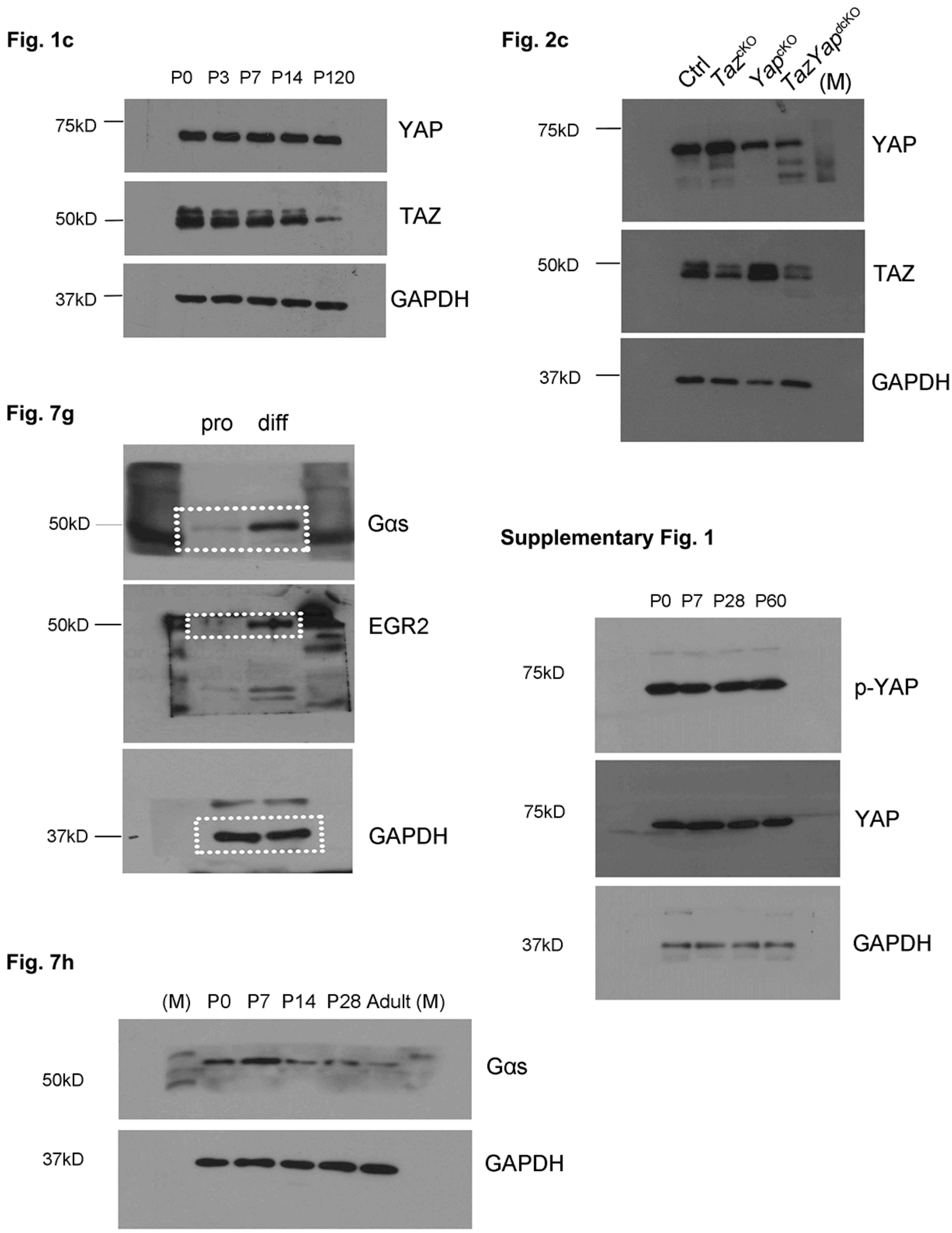


**Supplementary Fig. 9. Full scans of western blots**

Figure 1c, showing YAP and TAZ blot and GAPDH control in wildtype sciatic nerve at P0, P3, P7, P14 and P120;

Figure 2c, showing YAP and TAZ blot and GAPDH control in control and *Taz*$^{cKO}$, *Yap*$^{cKO}$, and *TazYap*$^{dcKO}$ sciatic nerves at P7;

Figure 7g, showing Gαs, EGR2 blot and GAPDH control in proliferating and

differentiated rat Schwann cells.

Figure 7h, showing Gαs blot and GAPDH control in wildtype sciatic nerve at P0, P7, P14, P28 and adult age.

Supplementary Figure 1, showing Phospho-YAP, YAP blot and GAPDH control in wildtype sciatic nerve at P0, P7, P28 and P60.

**Supplementary Table 1: Representative differentially expressed genes and pathways from RNA-seq between control and *Taz*/*Yap* mutant nerves**

| Clusters | Genes | Ctrl (FPKM) | $Taz^{cKO}Yap^{Het}$ (FPKM) | $TazYap^{dcKO}$ (FPKM) | Fold change (Ctr/$TazYap^{dcKO}$) |
|---|---|---|---|---|---|
| **Myelin genes** | *Mbp* | 10026.8 | 313.54 | 183.912 | 54.51955 |
| | *Prx* | 1220.42 | 105.604 | 30.2304 | 40.37062 |
| | *Mal* | 976.067 | 73.1745 | 30.9818 | 31.50453 |
| | *Pmp22* | 10674.9 | 459.52 | 346.16 | 30.83805 |
| | *Plp1* | 568.587 | 197.169 | 61.4021 | 9.260058 |
| **Myelination positive regulators** | *Egr2* | 82.8681 | 24.4902 | 8.37706 | 9.892265 |
| | *Sox10* | 195.744 | 76.5919 | 36.6179 | 5.345582 |
| | *Erbb3* | 86.9202 | 57.992 | 19.0434 | 4.564321 |
| | *Pou3f1* | 103.864 | 74.1815 | 32.6919 | 3.177056 |
| | *Zeb2* | 48.8144 | 26.3044 | 18.0652 | 2.702123 |
| | *Erbb2* | 31.3246 | 16.8728 | 12.3977 | 2.526646 |
| **Negative regulators** | *Hes1* | 12.5309 | 23.3381 | 30.4939 | 0.410931 |
| | *Egr1* | 10.8199 | 21.9623 | 42.2143 | 0.256309 |
| **Cell cycle related genes** | *Ccnc* | 8.86784 | 7.14211 | 5.88588 | 1.506629 |
| | *Ccng2* | 18.095 | 15.3328 | 13.5725 | 1.333211 |
| | *Ccnj* | 4.28313 | 3.94721 | 3.24877 | 1.318385 |
| | *Ccnk* | 14.4801 | 13.6385 | 11.8109 | 1.225995 |
| | *Cdk6* | 6.67158 | 3.06123 | 3.09898 | 2.152831 |
| | *Cdk7* | 10.115 | 8.90184 | 8.17581 | 1.237186 |
| | *Pim3* | 18.4193 | 11.4668 | 12.1354 | 1.517816 |
| | *Mycn* | 13.3193 | 12.4955 | 3.30641 | 4.028327 |
| **Hippo pathway** | *Ddah1* | 66.8666 | 15.1965 | 11.5185 | 5.805148 |
| | *Fgf1* | 46.396 | 8.67363 | 12.0326 | 3.855858 |
| | *Asap1* | 22.3732 | 15.4338 | 10.856 | 2.060906 |
| | *Agfg2* | 17.276 | 6.55884 | 8.96042 | 1.928035 |
| | *Amotl2* | 26.7045 | 18.0169 | 14.6402 | 1.824053 |
| | *Ect2* | 6.87661 | 4.56131 | 4.08803 | 1.682133 |
| | *Cenpf* | 6.95693 | 4.29089 | 4.14398 | 1.678804 |
| | *Fgf2* | 4.99452 | 2.90764 | 3.57072 | 1.398743 |
| **Laminin pathway** | *Itgb8* | 82.8255 | 38.2339 | 9.36512 | 8.84404 |
| | *Itga6* | 274.712 | 70.405 | 42.6792 | 6.436672 |
| | *Dag1* | 301.522 | 217.401 | 104.934 | 2.873444 |
| | *Itgb4* | 28.0649 | 8.56959 | 11.5922 | 2.421016 |
| | *Itga2* | 6.58964 | 3.33947 | 3.30509 | 1.993785 |
| | *Itga4* | 5.41787 | 3.41116 | 2.86193 | 1.893083 |
| | *Itgb1* | 223.235 | 202.159 | 183.721 | 1.215076 |

**Supplementary Table 2:**

**Primer sequences for qRT-PCR**

| gene | Species | Forward | Reverse |
|---|---|---|---|
| *Gapdh* | mouse | tccagtatgactctacccacg | cacgacatactcagcaccag |
| *Gli1* | mouse | acactcagctggactttgtggcta | agacactcatgttacccactgcca |
| *Gli2* | mouse | agagaagcagaagctatgcccaa | tgggcagcctccattctgttcata |
| *Gli3* | mouse | gctccaacatttccaacac | tgtgggcttgctctgtgagg |
| *Taz* | mouse | gtcaccaacagtagctcagatc | agtgattacagccaggttagaaag |
| *Yap* | mouse | gatccctgatgatgtaccactgcc | gccatgttgttgtctgatcgttgtg |
| *Gnas* | mouse | agcttccttaacgcccacc | ctcaggctcaacctcctcg |
| | | | |
| *Gapdh* | rat | tccagtatgactctacccacg | cacgacatactcagcaccag |
| *Taz* | rat | catggcggaaaaagatcctcc | gtcggtcacgtcataggactg |
| *Yap* | rat | gatccctgatgatgtaccactgcc | gccatgttgttgtctgatcgttgtg |
| *Gnas* | rat | ccgggatgagtttctgagaatc | caggtaaagtgagggtagcag |
| *Gnai1* | rat | ggtttacggacacatccatcat | gcctgcatattctggatagcat |
| *Gnaq* | rat | ggtcggggtactctgatga | acttgtatgggatcttgagagt |
| *Amotl2* | rat | ggagaagagttgcccacctat | tcgaagagcttcatcctgtcg |
| *Fgf1* | rat | ccctgaccgagaggttcatc | gtccctggtcccatccacg |
| *Ddah1* | rat | gcaatagggtccagcgaatct | gcttgtcataacgatggtcactc |
| *Mbp* | rat | ttgactccatcgggcgcttcttta | ttcatcttgggtcctctgcgactt |
| *Pmp22* | rat | catcgcggtgctagtgttg | aaggcggatgtggtacagttc |
| *Erbb2* | rat | accgacatgaagttgcgactc | aggtaagctccaaattgccct |
| *Erbb3* | rat | tgggttcgtgaactgtacca | ccggactgtcctgaaaacat |
| *Zeb2* | rat | acacacaggaaagagaccacac | cgcttgccacatttgtcacactga |
| *Ccnd1* | rat | gcgtaccctgacaccaatctc | acttgaagtaagaaacggagggc |
| *Cdk6* | rat | ccttacctcggtggtcgtc | gaacttccacgaaaaagaggct |
| *Ccnj* | rat | ttgaagagaaagaggacagcg | gttccactgaaaggtctctagg |
| *Pim3* | rat | cccgctggcgaaagaaattc | catttggcgaagcctccttta |
